# Agerarin, identified from *Ageratum houstonianum*, stimulates circadian CLOCK-mediated aquaporin-3 gene expression in HaCaT keratinocytes

**DOI:** 10.1038/s41598-017-11642-x

**Published:** 2017-09-11

**Authors:** Soon Young Shin, Da Hyun Lee, Ha-Na Gil, Beom Soo Kim, Jeong-Sook Choe, Jung-Bong Kim, Young Han Lee, Yoongho Lim

**Affiliations:** 1 Department of Biological Sciences, Sanghuh College of Life Sciences, Konkuk University, Seoul, 05029 Republic of Korea; 2 Cancer and Metabolism Institute, Konkuk University, Seoul, 05029 Republic of Korea; 3 Division of Bioscience and Biotechnology, BMIC, Konkuk University, Seoul, 05029 Republic of Korea; 4 Department of Agrofood Resources, National Academy of Agricultural Sciences, Rural Development Administration, Jeonju, 55365 Republic of Korea

## Abstract

The juice of *Ageratum houstonianum* is used in folk medicine as an external wound healing aid for skin injuries. However, the active component of *A. houstonianum* and its mode of action in skin wound healing has not been investigated. This study was conducted to investigate the effect of *A. houstonianum* ethanolnolic extract (AHE) on the expression of aquaporin-3 (AQP3), an integral membrane protein for water and glycerol transport in keratinocytes, and to identify the structure of the *A. houstonianum* bioactive compound. Here, we show that AHE increased *AQP3* gene expression at the transcriptional level through the p38 MAPK pathway in HaCaT cells. Furthermore, AHE ameliorated suppression of *AQP3* expression caused by ultraviolet B (UVB) irradiation. Agerarin (6,7-dimethoxy-2,2-dimethyl-2*H*-chromene) was identified as the bioactive compound responsible for the up-regulation of *AQP3* expression by enhancing the expression of the transcription factor circadian locomotor output cycles kaput (*CLOCK*). In conclusion, agerarin is a bioactive compound in AHE responsible for CLOCK-mediated *AQP3* expression in keratinocytes.

## Introduction

Water makes up about 60% of human body weight and plays an important role in maintaining tissue homeostasis in all biological physiologies. The skin contains about 30% of total body water and serves a waterproofing function for the body^1^. The skin is the soft, outer covering tissue and plays a key role in protecting the body against hazardous environmental threats, such as pathogens, external chemical insults, heat, and water loss. It is a highly organized multilayered epithelium, divided into two compartments: the epidermis and dermis. The epidermis is subdivided into several layers, including the stratum corneum, stratum granulosum, stratum spinosum, and stratum basale. The stratum corneum is located at the outermost part of the body and thereby functions as the primary permeability barrier, preventing water loss from the skin surface^2^.

Multiple complex factors are involved in the skin wound healing process. It is generally accepted that maintenance of skin hydration is important for the healing of various skin injuries^3^. Failure of skin water homeostasis causes delayed wound healing, hypertrophic scarring^4^, and development of various skin diseases, such as psoriasis^5^ and atopic dermatitis^6, 7^. Furthermore, a decrease in the hydration level of the epidermis directly up-regulates the expression of various pro-inflammatory cytokines, including interleukin (IL)-1β, IL-8, tumor necrosis factor-α, and matrix metalloproteinase 9, demonstrating that epithelial hydration status plays a critical role in optimizing wound healing^8^.

Aquaporins (AQPs) are integral pore proteins that transport water molecules across the membrane^9^. In humans, at least 13 AQPs (AQP0-AQP12) have been identified to date. There are two types of AQPs: water-selective transporters such as AQP0, AQP2, AQP4, and AQP5; and water and glycerol transporters such as AQP3, AQP7, and AQP9^9^. Of these, AQP3 is highly expressed in the plasma membranes of keratinocytes and functions as both a water and glycerol transporter (aquaglyceroporin) in the basal layer of the skin epidermis^2, 10^. AQP3-deficient mice have relatively dry skin and reduced skin elasticity as compared to wild-type mice^11^. A selective decrease in glycerol levels in AQP3-deficient mice may account for impaired skin hydration, elasticity, and barrier function^12^. In addition, AQP3 is believed to be important in wound healing by enhancing epidermal cell migration and proliferation^3^.

*Ageratum houstonianum*, a member of the Asteraceae family, is commonly known as flossflower, bluemink, blueweed, pussy foot, or Mexican paintbrush (Supplemental Fig. S1). It is a cool-season annual plant growing to a height of 0.3–1 m, with ovate to triangular leaves and blue (sometimes white, pink, or purple) flower heads (In: PlantNET, The NSW Plant Information Network System, Royal Botanic Gardens and Domain Trust, Sydney; http://plantnet.rbgsyd.nsw.gov.au org). The plant is apparently native to Central America and southeastern Mexico and has been cultivated and naturalized in North America, Africa, Asia, Europe and Oceania (In: Invasive Species Compendium, Wallingford, UK; http://www.cabi.org/isc). Various bioactive components have been isolated from the plant, including flavones, pyrrolizidine alkaloids, steroids, benzofuran, and precocenes^13–16^. It has been reported that *A. houstonianum* has broad biological activities, including antifungal, antibacterial, and antimicrobial activities^16–19^. It has been used for treating pain and infections, especially for healing of external wounds and skin diseases^15, 20^. However, the active component of *A. houstonianum* and its mode of action for curing skin wounds have not been investigated.

In this study, we aimed to investigate the effects of *A. houstonianum* ethanolic extract (AHE) on the expression of aquaporin-3 (AQP3). Our results demonstrate that agerarin can be used as a potential adjunct to enhance the treatment of various skin diseases by providing adequate hydration in the epidermis.

## Results

### AHE up-regulates *AQP3* expression in HaCaT keratinocytes

**Figure 1:**
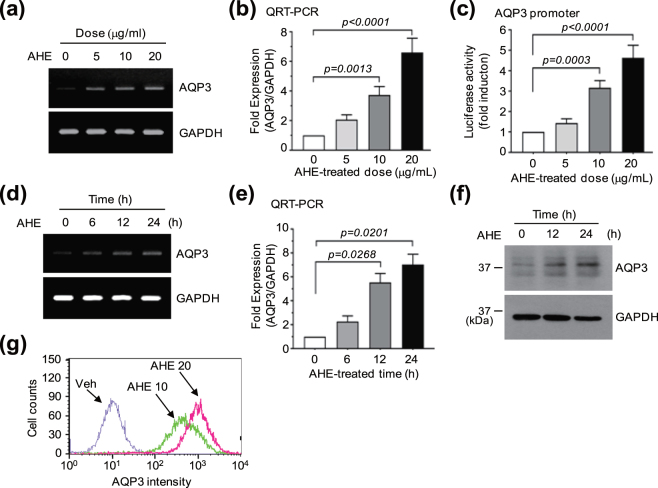
Effect of AHE on AQP3 expression. (**a**) HaCaT cells were treated with different concentrations of AHE for 24 h. AQP3 mRNA expression was analyzed by RT-PCR. *GAPDH* expression was used as an internal control. (**b**) HaCaT cells were treated with as in (**a**). *AQP3* mRNA levels were quantitated by qRT-PCR. *GAPDH* mRNA level was used for normalization. (**c**) *AQP3* promoter assay. pAQP3-Luc(−1090/+16) reporter was transfected into HaCaT cells. After 48 h, cells were treated with different concentrations of AHE. After 8–12 h, cells were collected and luciferase activities were measured. (**d**) HaCaT cells were treated with 20 μg/mL AHE for various time periods. *AQP3* mRNA expression was analyzed by RT-PCR. *GAPDH* expression was used as an internal control. (**e**) HaCaT cells were treated as in (**d**). *AQP3* mRNA levels were measured by qRT-PCR. The relative fold changes were normalized to the expression of *GAPDH* mRNA. (**f**) HaCaT cells were treated with 20 μg/mL AHE for various time periods. AQP3 protein levels were analyzed by immunoblotting. GAPDH expression was used as an internal control. (**g**) Flow cytometry. HaCaT cells were treated with AHE (10 and 20 μg/mL) and the percentage of AQP3 positive cells were measured by flow cytometry. Full-length gels (**a** and **d**) and blots (**f**) are presented in Supplemental Fig. S12.

To investigate whether AHE alters the expression of the *AQP3* gene, we first measured *AQP3* mRNA levels in AHE-treated HaCaT human immortalized keratinocytes. HaCaT cells were treated with different concentrations (0, 5, 10, and 20 μg/mL) of AHE, and levels of *AQP3* mRNA were measured by reverse transcription-polymerase chain reaction (RT-PCR) analysis. As shown in Fig. 1a, *AQP3* mRNA expression was substantially elevated by AHE treatment in a dose-dependent manner. Quantitative real-time PCR (qRT-PCR) analysis was conducted to precisely quantify the dose effect of AHE on *AQP3* mRNA expression. *AQP3* mRNA levels were significantly increased 3.7 ± 0.57-fold (*P* = 0.0013) and 6.6 ± 0.96-fold (*P* < 0.0001) after treatment of AHE at 10 and 20 μg/mL, respectively, compared with basal levels (Fig. 1b). To determine whether AHE-induced *AQP3* up-regulation occurred at the transcriptional level, an *AQP3* promoter reporter, pAQP3-Luc(−1090/+16), was generated and transfected into HaCaT keratinocytes. As shown in Fig. 1c, the luciferase reporter activity under control of the *AQP3* gene promoter was significantly (*P* < 0.001) elevated when the cells were treated with AHE (>10 μg/mL). A time-course experiment revealed that increases in *AQP3* mRNA were detectable as early as 6 h after AHE treatment, and levels gradually increased in a time-dependent fashion (Fig. 1d). qRT-PCR showed that *AQP3* mRNA levels were significantly increased 5.5 ± 0.75-fold (*P* = 0.0268) and 7.0 ± 0.86-fold (*P* = 0.0201) compared with basal levels after 12 and 24 h, respectively (Fig. 1e). To further evaluate whether AHE induced the accumulation of AQP3 proteins, immunoblot analysis was performed to detect proteins in the lysates of AHE-treated HaCaT keratinocytes. In accordance with RT-PCR analysis, treatment with AHE led to a time-dependent elevation in the amount of AQP3 protein (Fig. 1f). We confirmed AHE-induced expression of AQP3 proteins by flow cytometry. AHE dose-dependently elevated the population of AQP3-positive cells (Fig. 1g), suggesting that AHE-induced AQP3 proteins are located on the cell surface membrane. Thus, the *AQP3* gene is up-regulated at the transcriptional level by AHE treatment in HaCaT keratinocytes.

### Inhibition of p38 MAPK abrogates AHE-induced *AQP3* mRNA expression

**Figure 2:**
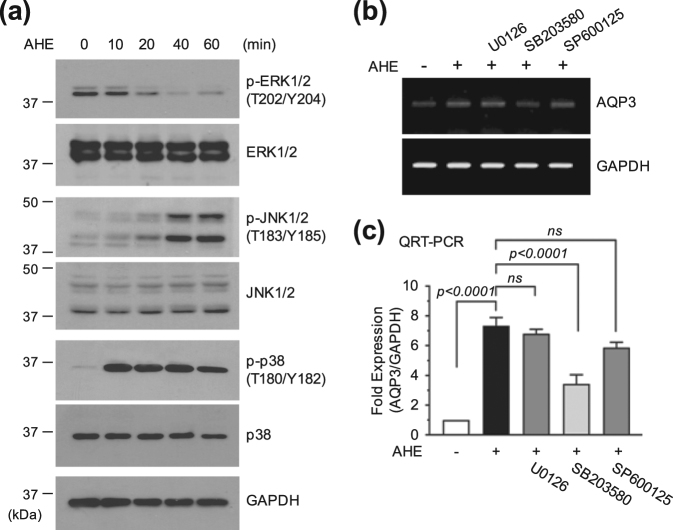
Role of MAPK signaling in AHE-induced *AQP3* expression. (**a**) HaCaT cells were treated with 20 μg/mL AHE for various lengths of time. Phosphorylation status of MAPKs were analyzed by immunoblotting. GAPDH antibody was used as an internal control to show equal protein loading. (**b**) HaCaT cells were either untreated or pretreated with U0126 (10 μM), SB203580 (20 μM), or SP600125 (25 μM) before addition of 20 μg/mL AHE for 24 h. *AQP3* mRNA expression was analyzed by RT-PCR. *GAPDH* expression was used as an internal control. (**c**) HaCaT cells were treated as in (**b**). *AQP3* mRNA levels were measured by qRT-PCR. *GAPDH* mRNA level was used for normalization. ns, not significant. Full-length blots (**a**) and gels (**b**) are presented in Supplemental Fig. S13.

Previous studies have demonstrated that *AQP3* expression is regulated by the mitogen-activated protein kinase (MAPK) pathway^21^. To gain insight into the regulatory mechanism controlling *AQP3* up-regulation in HaCaT cells, we examined the effect of AHE on the activation of three major MAPKs: ERK1/2, JNK1/2, and p38 MAPK. Serum-starved HaCaT cells were treated with 20 μg/mL AHE for various lengths of time, and MAPK activation was assessed using phospho-specific antibodies. We found that levels of phosphorylated ERK1/2 gradually decreased, whereas increased levels of phosphorylated JNK1/2 and p38 MAPK were detected within 10 min of AHE treatment (Fig. 2a). To assess the potential involvement of MAPK members in mediating AHE-induced *AQP3* upregulation, specific chemical inhibitors were utilized. Pretreatment of HaCaT cells with the p38 MAPK inhibitor SB203580 significantly (*P* < 0.0001) abrogated AHE-induced accumulation of *AQP3* mRNA, whereas the MAPK kinase 1/2 (MEK1/2) inhibitor U0126 and the JNK inhibitor SP600125 had little effect, as revealed by RT-PCR (Fig. 2b) and qRT-PCR (Fig. 2c). At present, although it is difficult to define the roles of the different MAPK pathways, it seems likely that p38 MAPK, at least in part, mediates AHE-induced *AQP3* mRNA expression.

### UVB irradiation reduces *AQP3* expression in HaCaT keratinocytes

**Figure 3:**
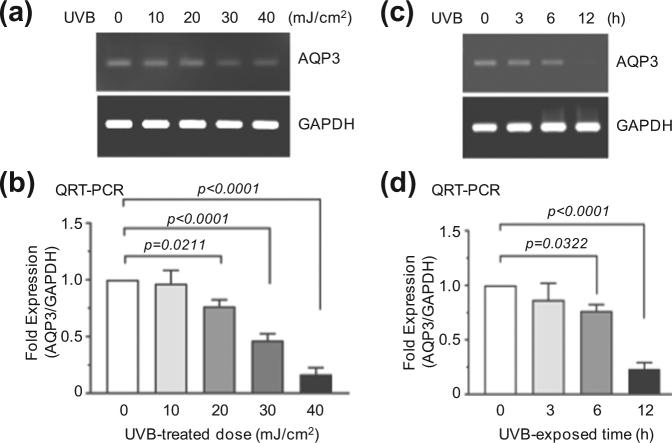
Effect of UVB on the suppression of AQP3 expression. (**a**) RT-PCR. HaCaT cells were irradiated with different doses of UVB for 24 h. *AQP3* mRNA expression was analyzed by RT-PCR. *GAPDH* expression was used as an internal control. (**b**) HaCaT cells were treated with as in (**a**). *AQP3* mRNA levels were measured by qRT-PCR. *GAPDH* mRNA level was used for normalization. (**c**) HaCaT cells were irradiated with UVB (30 mJ/cm^2^) for various time periods. *AQP3* mRNA expression was analyzed by RT-PCR. *GAPDH* expression was used as an internal control. (**d**) HaCaT cells were irradiated with UVB as in (**c**). *AQP3* mRNA levels were quantitated by qRT-PCR. The mRNA expression were normalized to the *GAPDH* mRNA. Full-length gels (**a** and **c**) are presented in Supplemental Fig. S14.

It has been reported that ultraviolet irradiation down-regulates *AQP3* expression^22^. UVB irradiation over 20 mJ/cm^2^ reduced *AQP3* mRNA expression in HaCaT cells, as revealed by RT-PCR (Fig. 3a). qRT-PCR showed that the relative expression of *AQP3* mRNA was significantly decreased 0.4 ± 0.1-fold and 0.18 ± 0.076-fold (all *P* < 0.0001) compared to levels in untreated controls at doses of 30 and 40 mJ/cm^2^, respectively (Fig. 3b). Time-course analysis showed that UVB-induced down-regulation of *AQP3* mRNA expression began to be detected after 6 h of UVB irradiation (30 mJ/cm^2^), as revealed by RT-PCR (Fig. 3c). Similar results were obtained by qRT-PCR (Fig. 3d). These data demonstrate that UVB irradiation at a dose of 30 mJ/cm^2^ for longer than 6 h down-regulates *AQP3* expression in HaCaT keratinocytes.

### AHE overcomes UVB-induced suppression of *AQP3* expression

**Figure 4:**
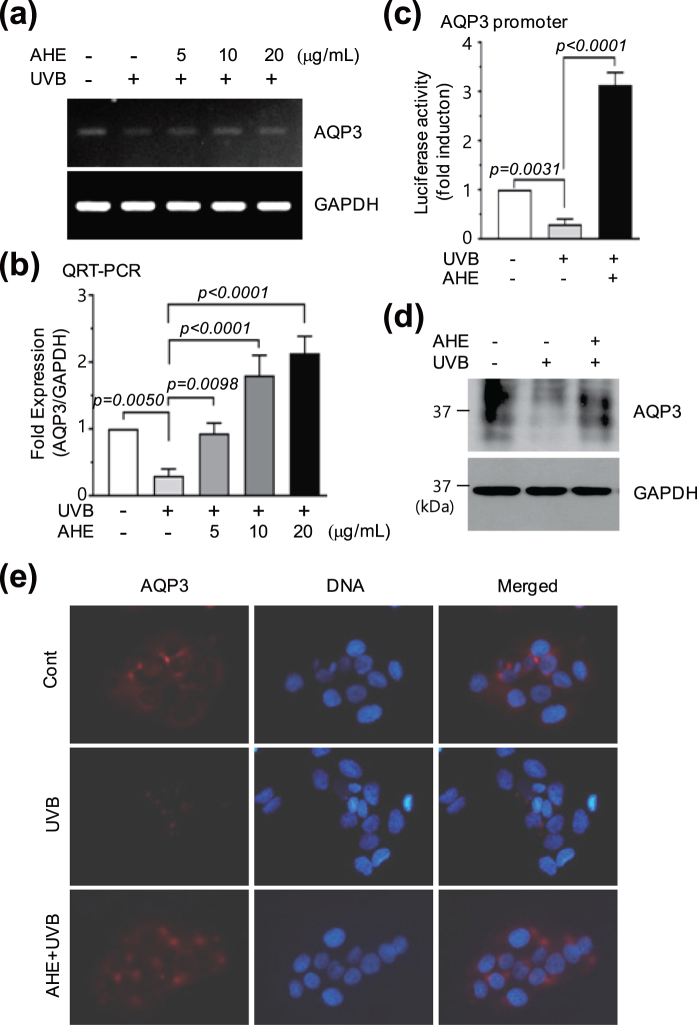
Effect of AHE on UVB-induced suppression of AQP3 expression. (**a**) HaCaT cells were irradiated with UVB (30 mJ/cm^2^) in the absence or presence of different concentrations of AHE. After 24 h, *AQP3* mRNA expression was analyzed by RT-PCR. *GAPDH* expression was used as an internal control. (**b**) HaCaT cells were treated as in (**a**) and qRT-PCR was performed. *GAPDH* mRNA level was used for normalization. (**c**) HaCaT cells were transfected with 0.2 μg of promoter reporter pAQP3-Luc(−1090/+16), followed by irradiation with UVB (30 mJ/cm^2^) in the absence or presence of 20 μg/mL AHE at 24 h post-transfection. After 8–12 h, luciferase activities were measured. (**d**) HaCaT cells were irradiated with UVB (30 mJ/cm^2^) in the absence or presence of AHE (20 and 10 μg/mL). After 24 h, AQP3 protein level was detected by immunoblotting. GAPDH was used as an internal control. (**e**) HaCaT cells cultured on a coverglass were treated as in (**d**). AQP3 antibody was incubated for 2 h, followed by AlexaFluor 555-conjugated (red signal) secondary antibody for additional 30 min. Nuclear DNA was stained with 0.1 μg/mL Hoechst 33258 (blue signal) for 10 min. Fluorescence-positive cells were examined under an EVOSf1^®^ fluorescence microscope. Full-length gels (**a**) and blots (**d**) are presented in Supplemental Fig. S15.

We next attempted to determine whether AHE has the ability to ameliorate ultraviolet B (UVB)-induced suppression of *AQP3* expression. HaCaT cells were treated with AHE for 30 min, followed by irradiation with UVB (30 mJ/cm^2^). As shown in Fig. 4a, UVB-induced suppression of *AQP3* mRNA expression was gradually ameliorated by AHE treatment. qRT-PCR showed that levels of *AQP3* mRNA significantly (*P* < 0.0001) increased about 2-fold compared with basal levels following pretreatment with >10 μg/mL AHE (Fig. 4b). Moreover, UVB-induced suppression of both *AQP3* promoter activity (Fig. 4c) and AQP3 protein accumulation (Fig. 4d) was inhibited by AHE treatment. We confirmed the effect of AHE on the accumulation of AQP3 protein by immunofluorescence microscopy. HaCaT cells cultured on a coverglass were pretreated with AHE before UVB irradiation. As shown in Fig. 4e, the reduction in AQP3 protein staining following UVB irradiation was inhibited by AHE treatment. These results suggest that AHE is able to overcome UVB-induced suppression of *AQP3* expression.

### Identification of agerarin as an active compound of AHE

**Figure 5:**
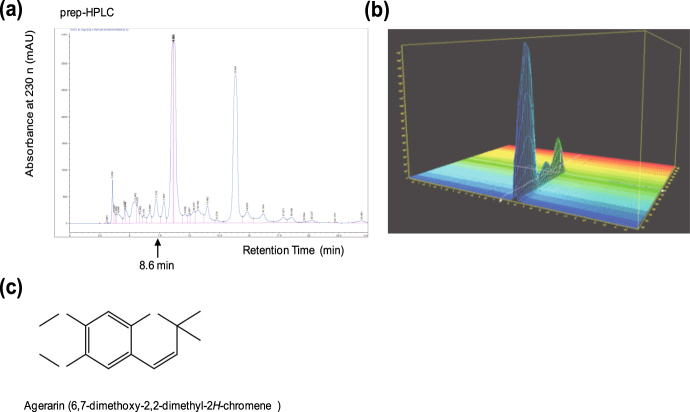
Identification of agerarin as an active component of AHE. (**a**) Chromatogram of agerarin at 230 nm. (**b**) The peak detected at 8.6 min on the HPLC chromatogram with photodiode array detector at 230 nm. (**c**) The structure of agerarin, 6,7-dimethoxy-2,2-dimethyl-2*H*-chromene.

To identify the active component responsible for *AQP3* up-regulation, AHE was further fractionated using n-hexane, chloroform, and water (Supplemental Fig. S2a). We observed that n-hexane fraction (AHE-Hx) significantly (*P* < 0.0001) inhibited UVB-induced suppression of *AQP3* expression (Supplemental Fig. S2b) and was able to induce *AQP3* mRNA expression (Supplemental Fig. S2c), suggesting that AHE-Hx contains active components responsible for the induction of *AQP3*. AHE-Hx fraction was further separated by a prep-high-performance liquid chromatography (prep-HPLC) with a Luna C18 column. As shown in Fig. 5a, a major peak was observed at 8.6 min in the chromatogram. The peak (named AG-H1) was a single compound as revealed by analysis with a photodiode array detector (Fig. 5b). To elucidate the chemical structure, NMR spectroscopy was carried out using a Bruker AVANCE 400 spectrometer system. Twelve peaks were observed in the ^13^C nuclear magnetic resonance (NMR) spectrum (Supplemental Fig. S3), and seven peaks were observed in the ^1^H NMR spectrum (Supplemental Fig. S4). Distortionless Enhancement by Polarization Transfer (DEPT) data showed that this compound consists of three quartet, four doublet, and five singlet carbons (Supplemental Fig. S5). Since two carbon peaks at 56.1 and 56.7 ppm were attached directly to the proton peaks at 3.80 and 3.78 ppm, respectively, in the heteronuclear multiple quantum coherence (HMQC) spectrum (Supplemental Fig. S6), they were assigned two methoxy groups. The proton peak at 1.38 ppm attached directly to the carbon peak at 27.8 ppm was assigned a methyl group, and they were determined to be two methyl groups based on the integration value of the proton peak. Nine carbon peaks and four proton peaks showed the pattern of the chromene scaffold^23^. Based on interpretation of the total correlated spectroscopy (TOCSY; Supplemental Fig. S7), correlated spectroscopy (COSY; Supplemental Fig. S8), heteronuclear multiple bonded connectivities (HMBC; Supplemental Fig. S9), and heteronuclear multiple quantum coherence (HMQC) spectra, this scaffold was identified as a 2*H*-chromenone. The important correlations obtained from the interpretations of the COSY and HMBC spectra are provided as Supplemental Fig. S10. The complete assignments of the ^1^H and ^13^C NMR data are listed in Supplemental Table S1. As a result, the AG-H1 peak was identified as 6,7-dimethoxy-2,2-dimethyl-2*H*-chromene (PubChem CID: 12565; named agerarin; Fig. 5c). Agerarin is known as ageratochromene^24^ or precocene II^25^. To confirm this structure, High resolution mass spectrometry (HR/MS) was carried out with ultra-performance liquid chromatography-hybrid quadrupole/time-of-flight mass spectrometry (UPLC-Q-Tof-MS). The data were collected as M + H ions as shown in Supplemental Fig. S11. The calculated mass was 221.1178, and the mass found by HR/MS was 221.5345. These data demonstrate that the agerarin structure determined by NMR spectroscopy agreed with that obtained by HR/MS.

### Agerarin alone induces *AQP3* mRNA expression

**Figure 6:**
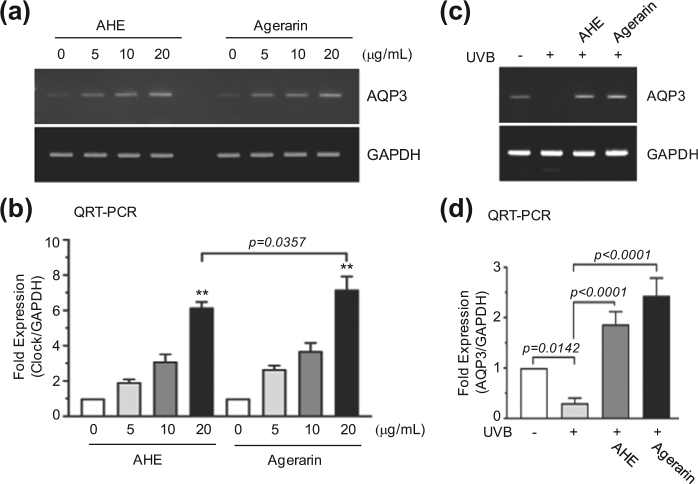
Effect of agerarin on the expression of *AQP3*. (**a**) HaCaT cells were treated with different concentrations of AHE or agerarin. After 24 h, *AQP3* mRNA expression was analyzed by RT-PCR. *GAPDH* expression was used as an internal control. (**b**) HaCaT cells were treated as in (**a**) and *AQP3* mRNA levels were measured by qRT-PCR. The relative fold changes were normalized to the expression of *GAPDH* mRNA. **p < 0.0001 compared to vehicle control (0 μg/mL). (**c**) HaCaT cells were irradiated with UVB (30 mJ/cm^2^) in the absence or presence of AHE or agerarin (each 20 μg/mL). After 24 h, *AQP3* mRNA expression was analyzed by RT-PCR. *GAPDH* expression was used as an internal control. (**d**) HaCaT cells were treated as in (**c**) and qRT-PCR was performed. *GAPDH* mRNA level was used for normalization. Full-length gels (**a** and **c**) are presented in Supplemental Fig. S16.

We next explored whether agerarin alone increases *AQP3* expression. RT-PCR analysis shows that treatment with agerarin alone markedly increased *AQP3* mRNA levels, comparable in magnitude to the increase with AHE treatment (Fig. 6a). qRT-PCR demonstrates that both AHE and agerarin increased *AQP3* mRNA levels in a dose-dependent manner (Fig. 6b). At the highest dose (20 μg/mL), AHE and agerarin significantly increased *AQP3* mRNA levels by 6.27-fold and 7.17-fold, respectively, compared to vehicle control (*p* < 0.0001). There was a statistically significant difference between AHE and agerarin treated groups (*P* = 0.0357 by Sidak’s mutiple comparisons test, n = 3). Furthermore, agerarin alone inhibited UVB-induced suppression of *AQP3* mRNA expression, as revealed by RT-PCR (Fig. 6c). Similar results were obtained by qRT-PCR (Fig. 6d). These results demonstrate that agerarin isolated from AHE is responsible for the up-regulation of *AQP3* expression.

### Agerarin stimulates the circadian *CLOCK* gene expression

**Figure 7:**
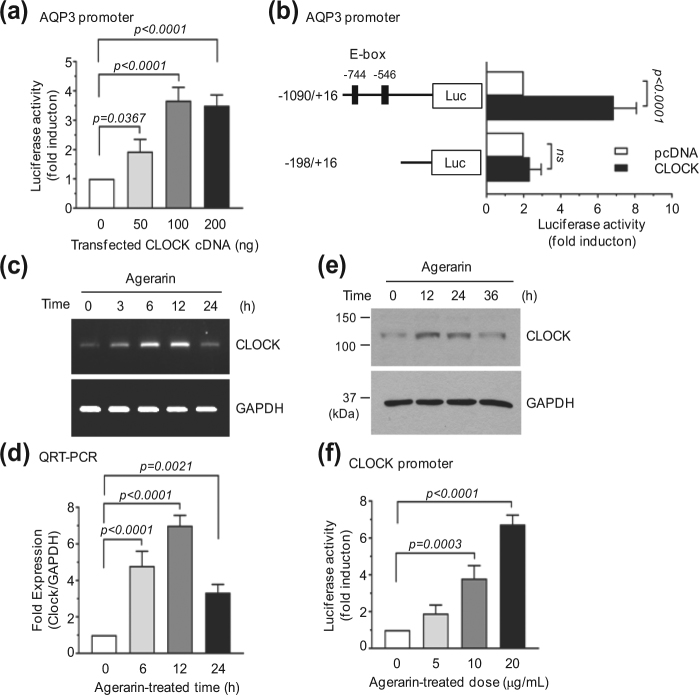
Effect of agerarin on the expression of *CLOCK*. (**a**) HaCaT cells were transfected with 0.2 μg of promoter construct pAQP3-Luc(−1090/+16) along with different concentrations of expression plasmids for *CLOCK*, pcDNA3.1/Clock. After 24 h, cells were collected and assayed for luciferase activity. (**b**) HaCaT cells were transfected with 0.2 µg of a full-length (−1090/+16) or 5′-deletion construct (−198/+16) along with 0.1 μg of empty vector (pcDNA3,1) or pcDNA3.1/Clock plasmids, and measured luciferase activity after 24 h. (**c**) HaCaT cells were treated with agerarin (20 μg/mL) for various time periods. *AQP3* mRNA expression was analyzed by RT-PCR. *GAPDH* expression was used as an internal control. (**d**) HaCaT cells were treated as in (**b**) and *AQP3* mRNA levels were measured by qRT-PCR. *GAPDH* mRNA level was used for normalization. (**e**) HaCaT cells were treated with 20 μg/mL agerarin for various time periods. AQP3 protein level was detected by immunoblotting. GAPDH was used as an internal control. (**f**) *CLOCK* promoter assay. HaCaT cells were transfected with 0.2 μg of promoter reporter pClock-Luc(−1000/−1). After 48 h, HaCaT cells were treated with different concentrations of agerarin. After 12 h, luciferase activities were measured. Full-length gels (**c**) and blots (**e**) are presented in Supplemental Fig. S17.

To investigate the molecular mechanism underlying AHE-induced *AQP3* expression, we utilized agerarin. A previous report demonstrated that *AQP3* expression in human keratinocytes is regulated by molecular clock genes, including *Circadian Locomotor Output Cycles Kaput* (CLOCK)^26^. On the basis of this study, we considered the possibility that molecular clock genes play an important role in agerarin-induced transcriptional activation of the *AQP3* gene. To test this possibility, we first examined whether CLOCK is required for the induction of *AQP3* gene promoter activity. HaCaT cells were transfected with the *AQP3* promoter reporter, pAQP3-Luc(−1090/+16), along with an expression plasmid for *CLOCK*. In accordance with a previous report^26^, transient transfection of the *CLOCK* gene resulted in stimulation of promoter reporter activity in a plasmid concentration-dependent manner (Fig. 7a). Moreover, a 5′-deletion construct (−198/+16) that lacks CLOCK binding sites (E-box) resulted in complete loss of CLOCK-induced promoter activation (Fig. 7b). These data suggest that CLOCK plays an important role in AQP3 transcriptional activation. A time-course experiment showed that levels of *CLOCK* mRNA increased within 3 h, were maintained through 12 h, and then decreased at 24 h after the addition of agerarin (Fig. 7c). qRT-PCR analysis revealed that agerarin-induced *CLOCK* mRNA levels were significantly increased 4.8 ± 0.79-fold (*P* < 0.0001), 7.0 ± 0.57-fold (*P* < 0.0001), and 3.3 ± 0.45-fold (*P* = 0.0021) compared with basal levels after 6, 12, and 24 h, respectively (Fig. 7d). Immunoblot analysis showed similar results (Fig. 7e). To determine whether agerarin-induced *CLOCK* up-regulation occurred at the transcriptional level, a *CLOCK* promoter reporter, pClock-Luc(−1000/+47), was generated and transfected into HaCaT keratinocytes. As shown in Fig. 7f, treatment with agerarin at 5, 10, and 20 μg/mL resulted in 1.9 ± 0.46-fold (*P* > 0.05), 3.8 ± 0.7-fold (*P* = 0.0003), and 6.7 ± 0.50-fold (*P* < 0.0001) increases, respectively, in luciferase reporter activity under the *CLOCK* gene promoter. These results demonstrate that *CLOCK* expression is up-regulated by agerarin isolated from AHE and is involved in the transcriptional activation of the *AQP3* gene in HaCaT keratinocytes.

### Silencing of *CLOCK* abrogates agerarin-induced *AQP3* upregulation

**Figure 8:**
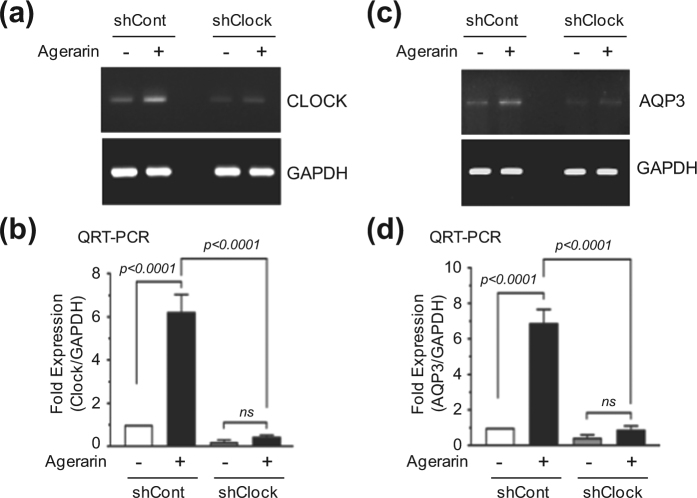
Effect of *CLOCK* silencing on agerarin-induced *AQP3* expression. (**a**) HaCaT/shCont and HaCaT/shClock cells were treated with agerarin (each 20 μg/mL) for 12 h. *CLOCK* mRNA expression was analyzed by RT-PCR. *GAPDH* expression was used as an internal control. (**b**) HaCaT cells were treated as in (**a**) and *CLOCK* mRNA levels were measured by qRT-PCR. *GAPDH* mRNA level was used for normalization. (**c**) HaCaT/shCont and HaCaT/shClock cells were treated with agerarin (20 μg/mL). After 24 h, *AQP3* mRNA expression was analyzed by RT-PCR. *GAPDH* expression was used as an internal control. (**d**) HaCaT cells were treated as in (**c**) and *AQP3* mRNA levels were measured by qRT-PCR. *GAPDH* mRNA level was used for normalization. Full-length gels (**a** and **c**) are presented in Supplemental Fig. S18.

To corroborate the functional role of CLOCK in agerarin-induced *AQP3* expression, we established HaCaT variant cell lines expressing scrambled control shRNA (shCont) or *CLOCK* shRNA (shClock), using a lentiviral expression system. Stable knockdown of *CLOCK* expression was verified by RT-PCR. We further confirmed the silencing of *CLOCK* expression after agerarin treatment for 12 h, by RT-PCR (Fig. 8a) and qRT-PCR (Fig. 8b). We next addressed whether *AQP3* expression is affected by the silencing of *CLOCK* expression. HaCaT cells were treated with agerarin for 24 h, and then *AQP3* mRNA levels were measured by RT-PCR. Agerarin-induced *AQP3* expression was markedly reduced in HaCaT/shClock cells compared with that in HaCaT/shCont cells (Fig. 8c). qRT-PCR showed that agerarin-induced *AQP3* mRNA expression was significantly (*P* < 0.0001) inhibited by the silencing of *CLOCK* (Fig. 8d). These results strongly implicate CLOCK as the transcription factor responsible for agerarin-induced *AQP3* mRNA expression.

## Discussion

The juice of *A. houstonianum* has been used in folk medicine for external wound healing of skin injuries in Korea, Mexico, India, and Nepal^15, 20^. However, the mode of action of *A. houstonianum* in skin wound healing had not been investigated. Hydration status in the epidermis is important for maintenance of the skin barrier structure and the healing process following various skin injuries^27^. Aquaglyceroporin AQP3 plays an essential role in transporting both water and glycerol in the epidermal basal layer^10^. In this study, we evaluated the effect of *A. houstonianum* ethanolic extract (AHE) on the expression of *AQP3* in human immortalized HaCaT keratinocytes. In addition, we identified agerarin as a bioactive component of AHE responsible for *AQP3* up-regulation. To our knowledge, this is the first report showing the effects of AHE on *AQP3* up-regulation in keratinocytes.

In the skin, AQP3 expressed on epidermal keratinocytes transports both water and glycerol from the dermis to the epidermis, thereby maintaining proper hydration levels in the epidermis. Glycerol is a substrate for the synthesis of triglycerides and various lipids in the living body. It has been demonstrated that AQP3-facilitated glycerol plays a critical role in the maintenance of skin hydration as well as in lipid metabolism and wound healing^11^. In this study, we found that AHE significantly elevated *AQP3* mRNA expression in dose- and time-dependent manners. To determine whether AHE-induced *AQP3* up-regulation occurred at the transcriptional level, we isolated the 5′-regulatory region of the human *AQP3* gene located within 1.1 kb upstream of the transcriptional start site and subcloned this into a luciferase reporter vector, yielding pAQP3-Luc(−1090/+16). *AQP3* gene promoter activity was significantly elevated by treatment with >10 μg/mL AHE, suggesting that AHE stimulates expression of the *AQP3* gene at the transcriptional level. We next identified the chemical structure of the active component of AHE, 6,7-dimethoxy-2,2-dimethyl-2*H*-chromene (named agerarin) as revealed by ^1^H and ^13^C NMR spectrometry and confirmed that agerarin is able to stimulate *AQP3* gene transcription.

Skin hydration is controlled by circadian rhythms^28^. The circadian clock is a highly conserved molecular timing system that coordinates various metabolic and physiological processes within a period of ~24 h. In mammals, central circadian rhythms are regulated by a highly specialized master pacemaker located in the suprachiasmatic nucleus of the anterior hypothalamus, which receives and conveys environmental timing information to the local organs^29^. Peripheral tissue clocks also exist and are involved in the regulation of local metabolism and physiology^30, 31^. Indeed, a recent study demonstrates that a natural flavone called Nobiletin acts on the circadian clock to enhance glucose metabolism^32^, suggesting that pharmacological modulation of circadian clock can regulate physiological responses. Both central and peripheral circadian clocks are regulated by circadian transcription factors, such as CLOCK and BMAL1 (brain and muscle aryl hydrocarbon receptor nuclear translocator-like protein-1)^29, 33–35^. Circadian clock genes are also expressed in the human skin^36^ and are involved in temperature control and cholesterol metabolism^37^. AQP3 expression oscillates with a period length of about 24 h, which is regulated by the CLOCK/BMAL1 circadian proteins in HaCaT keratinocytes^26^. In an effort to understand the molecular mechanism by which the *AQP3* gene is regulated by agerarin, we tested whether the circadian gene *CLOCK* is involved in agerarin-induced *AQP3* up-regulation. Our data showed that agerarin treatment caused the accumulation of *CLOCK* mRNA and protein levels within 6 h and 12 h, respectively. Promoter reporter assay revealed that agerarin-induced up-regulation of *CLOCK* is also regulated at the transcriptional level. As forced expression of CLOCK stimulated *AQP3* promoter reporter activity, we hypothesized that agerarin-induced CLOCK targets the *AQP3* gene in HaCaT keratinocytes. To corroborate the functional role of CLOCK in agerarin-induced *AQP3* expression, we established HaCaT variant cell lines expressing scrambled control shRNA (HaCaT/shCont) or *CLOCK* shRNA (HaCaT/shClock), using a lentiviral expression system. Our data showed that stable silencing of *CLOCK* expression significantly attenuated agerarin-induced *AQP3* expression, supporting the notion that CLOCK is directly involved in agerarin-induced *AQP3* expression. Although further studies regarding the interactive mechanisms of agerarin-induced *CLOCK* transcription are required, our results demonstrate that agerarin enhances *AQP3* gene expression through the activation of the circadian CLOCK protein.

MAPK signaling pathways, including ERK1/2, JNK1/2, and p38 MAPK, are well-known intracellular pathways that provide a mechanism for controlling the expression of tissue-specific target genes. Importantly, MAPK pathways are involved in the regulation of the circadian clock^38^. Our data showed that AHE rapidly induces the phosphorylation of JNK1/2 and p38 MAPK but not ERK1/2. We also observed that treatment with the p38 kinase inhibitor SB203580 but not with the JNK inhibitor SP600125 effectively inhibited AHE-induced *AQP3* mRNA expression. These data suggest that the p38 MAPK signaling pathway may be involved in AHE-induced *AQP3* expression in HaCaT keratinocytes. The role of p38 MAPK in regulating circadian rhythms in chicken^39^ and fruit fly^40^ has been demonstrated. The p38 MAPK pathway is also involved in TGFβ1-induced *AQP3* up-regulation in human peritoneal mesothelial cells^41^. Thus, it is possible that agerarin-induced *AQP3* up-regulation is mediated by p38 MAPK-dependent CLOCK expression. It would be interesting to address the role of p38 MAPK in the regulation of CLOCK-mediated *AQP3* expression in future studies.

In conclusion, this study demonstrated that the ethanolic extract of *A. houstonianum* (AHE) has pharmacological activity, enhancing expression of the aquaglyceroporin *AQP3* in HaCaT keratinocytes. We also showed that agerarin is a bioactive component of AHE, responsible for circadian CLOCK-mediated up-regulation of *AQP3*. Further work will be needed to examine the therapeutic potential and side effects of agerarin by using animal models of skin wounds to confirm the effects seen in the *in vitro* experiments presented here.

## Methods

### Preparation of *A*. *houstonianum* extracts

Young plants of *A*. *houstonianum* were purchased from Yangjae flower market (Korea Agro-Fisheries & Food Trade, Seoul 06749, Republic of Korea) and taxonomically identified by a galenical pharmacist, Dr. Hi Jae Cho (Bio/Molecular Informatics Center, Konkuk University, Seoul, Republic of Korea). They were cultivated in flowerpots for three months in Konkuk University (Seoul, Republic of Korea), as shown in Supplemental Fig. S1. A voucher specimen was deposited at the College of Biological Science and Technology, Konkuk University, Republic of Korea. Air-dried *A*. *houstonianum* (1248 g) was immersed in ethanol for 3 days. The filtered solution was dried using a rotary evaporator under a reduced pressure, and the ethanolic extract (150.9 g, 12.09%) was obtained. This ethanolic extract (120 g) was used for liquid–liquid separation. Three fractions, including ethanol, n-hexane (17.63 g, 14.69%), chloroform (6.7 g, 5.58%), and water (93.01 g, 77.51%) were collected. Each fraction was dried using a freeze-dryer and dissolved in DMSO at 20 mg/mL.

### Preparative high performance liquid chromatography (prep-HPLC)

A prep-HPLC was carried out using an Agilent 1100 instrument with a Luna C18 column (10 × 250 mm, ø = 5 µm). The mobile phase was a mixture of acetonitrile/methanol/water (v/v 30:50:20), and the flow rate and injection volume were 3.0 mL/min and 50 µL, respectively. The eluents were detected at 230 nm, using a photodiode array detector. The samples were dissolved in isopropanol.

### Nuclear magnetic resonance (NMR) spectroscopy and mass spectrometry (MS)

For identification of the chemical structure, the following experiments were performed on a Bruker Avance 400 NMR spectrometer (9.4 Tesla; Karlsruhe, Germany): 1D-NMR experiments including ^1^H NMR, ^13^C NMR, distortionless enhancement by polarization transfer, and 2D-NMR experiments including COSY, TOCSY, HMQC, and HMBC. The sample was dissolved in CDCl_3_ and transferred into a 2.5-mm NMR tube. Detailed experimental procedures were described previously^42^. HR/MS was carried out on a Waters ACQUITY UPLC system (Waters, Milford, MA, USA) with UPLC-Q-Tof-MS as described previously^43^.

### Cell culture and reagents

HaCaT cells were obtained from the Cell Lines Service (Eppelheim, Germany) and were maintained in Dulbecco’s modified Eagle’s medium (DMEM) supplemented with 10% fetal bovine serum (Hyclone, Logan, UT, USA). Antibodies specific for phospho-ERK1/2 (Thr202/Tyr204), phospho-JNK1/2 (Thr183/Tyr185), and phospho-p38 MAP kinase (Thr180/Tyr182) were purchased from Cell Signaling Technology (Beverly, MA, USA). GAPDH antibody was obtained from Santa Cruz Biotechnology (Santa Cruz, CA, USA). Aquaporin-3 (AQP3) antibody was purchased from BOSTER (Pleasanton, CA, USA). An Alexa Fluor 555-conjugated secondary antibody was obtained from Invitrogen (Carlsbad, CA, USA). The Dual-Glo^®^ Luciferase Assay System for firefly and *Renilla* luciferase activities was purchased from Promega (Madison, WI, USA). Pierce^TM^ BCA Protein Assay Reagent was obtained from Thermo Scientific (Rockford, IL, USA). Other chemicals were purchased from Sigma-Aldrich (St. Louis, MO, USA). Plasmid clone of human CLOCK cDNA in the pBluescriptR cloning vector (clone ID:IRAK074P05) was provided by the RIKEN BioResource Center (Tsukuba, Ibaraki, Japan) through the National Bio-Resource Project of the MEXT, Japan.

### Reverse transcription-polymerase chain reaction (RT-PCR) and quantitative real-time PCR (qRT-PCR)

RT-PCR was performed as described previously^44^. Briefly, reverse transcription was performed using 1 μg of total RNA as a template and oligo(dT) primers. The resulting cDNA was subjected to PCR analysis, using gene-specific primers for *AQP3* (forward, 5′-CCTTTGGCTTTGCTGTCACTCT-3′; reverse, 5′-CGGGGTTGTTGTAGGGGTCA-3′), *CLOCK* (forward, 5′-TAGGGTATTTGCCATTTGA-3′; reverse, 5′-GCCAAGTTCTCGTCGTC-3′), and glyceraldehyde-3-phosphate dehydrogenase (*GAPDH*; forward, 5′-ACCCACTCCTCCACCTTTG-3′; reverse, 5′-CTCTTGTGCTGCTGGG-3′). PCR conditions were as follows: denaturation, 94 °C for 30 s; annealing, 55 °C for 30 s; elongation, 72 °C for 1 min. The amplified products were subjected to electrophoresis on a 1% agarose gel.

For qRT-PCR, a TaqMan-iQ supermix kit (Bio-Rad) was used according to the manufacturer’s instructions. TaqMan^TM^ fluorogenic probes were designed by Metabion International (Martinsried, Germany). The sequences of primers for qRT-PCR were as follows: AQP3 (forward, 5′-CTTGAGCATCCACTGACT-3′; reverse, 5′-GGGTGAGGGTAGATAGGG-3′; TaqMan probe, 5′-6-FAM-CCCTTCACGATCCACCCTTTCA-BHQ-3′), CLOCK (forward, 5′-CCATCTAGTATGCCACAA-3′; reverse, 5′-TGACCTTGAGAAAATCTTA-3′; TaqMan probe, 5′-FAM-CAGTAACTACATTCACTCAGGACAGGC-BHQ-1-3′), and GAPDH (forward, 5′-TCGACAGTCAGCCGCATCTTC-3′; reverse, 5′-CGCCCAATACGACCACCTCCG-3′; TaqMan probe, 5′-Yakima Yellow TM-CGTCGCCAGCCGAGCCACATCGC-BHQ-1-3′). The relative mRNA expression was analyzed after normalization to *GAPDH* mRNA using the software program provided by the manufacturer. The data shown represent the mean ± SD (*n* = 3).

### Immunoblot analysis

Immunoblot analysis was carried out as described previously^45^. Cell lysates (20 μg each) were separated by 10% SDS-polyacrylamide gel electrophoresis and transferred to nitrocellulose filters. The blots were then incubated with primary antibodies and developed with an enhanced chemiluminescence detection system (GE Healthcare Life Sciences, Piscataway, NJ, USA).

### Flow cytometry

Expression of the cell surface AQP3 protein was measured by flow cytometry. AQP3 protein was detected using 1:500 diluted ant-AQP3 antibody (BOSTER). The secondary antibody was 1:500 diluted Rhodamin Red X-conjugated anti-rabbit IgG (H + L) antibody (Jackson ImmunoResearch, West Grove, PA, USA). After washing three times with 5% bovine serum albumin in PBS, fluorescent intensities were measured on a BD FACS-Calibur (BD Biosciences, San Diego, CA, USA) using the FACSort program.

### Immunofluorescence microscopy

HaCaT keratinocytes were cultured on coverslips and treated with either vehicle (DMSO) or AHE (20 μg/mL) for 30 min, followed by irradiation with ultraviolet B (UVB; 30 mJ/m^2^) for 24 h. Immunofluorescence analysis was performed as described previously^46^. Primary antibody specific to AQP3 was incubated for 2 h followed by addition of Alexa-Fluor 555-conjugated secondary antibody (red signal) and further incubated for 1 h. Nuclear DNA (blue signal) was stained with 0.1 μg/mL Hoechst 33258 (Sigma-Aldrich) for 10 min. Staining was examined under an EVOS FL fluorescence microscope (Advanced Microscopy Group; Bothell, WA, USA).

### Construction of *AQP3* and *CLOCK* gene promoter reporters

Amplification of the 5′-flanking regions of the *AQP3* and *CLOCK* genes was performed by PCR, using Dr. Taq DNA Polymerase (Doctor protein, Seoul, Korea). The *AQP3* gene was amplified from pGL4-phAQP (RDB07311, Riken DNA bank, Japan), and the *CLOCK* gene was amplified from the genomic DNA of MDA-MB-231 cells. Nucleotides upstream of the transcription start site were numbered beginning at +1. An *AQP3* promoter fragment spanning positions −1090 to +16 was synthesized using the primers 5′-GGTGAATCCCCATCTCCACT-3′ (forward) and 5′-CCTTTATAGGAGCGCTGGAG-3′ (reverse, +16R). The amplified PCR products were ligated into the *Hind*III sites of the pGL4.17 vector (Promega), yielding pAQP3-Luc(−1090/+16). A deletion fragment of *AQP3* promoter was synthesized by PCR using the pAQP3-Luc(−1090/+16) plasmid as a template. Forward primer 5′-CTTGACGTCCCCTCCCTT-3′ and +16R reverse primer were used to generate deletion constructs. The amplified PCR products were ligated into the pGL4-basic vector, yielding pAQP3-Luc(−198/+16). A *CLOCK* promoter fragment spanning positions −1000 to +47 was synthesized using the primers 5′-TTTACGGCCAGAAAAGTGCATT-3′ (forward) and 5′-TTTACGGCCAGAAAAGTGCATT-3′ (reverse). The amplified PCR products were ligated into the *Kpn*I and *Bgl*II sites of the pGL4.17 vector (Promega), yielding pClock-Luc(−1000/+47). The resultant constructs were verified by DNA sequencing (Macrogen, Seoul, Korea).

### Transient transfection and promoter reporter assays

HaCaT cells seeded onto 12-well plates were transfected with *AQP3* or *CLOCK* promoter construct (0.2 μg), using Lipofectamine 2000 reagent (Invitrogen Life Technologies, San Diego, CA, USA) according to the manufacturer’s instructions. Where indicated, CLOCK expression plasmid (pcDNA3.1/Clock) was also included. Luciferase activities were measured using the Dual-Glo^®^ Luciferase Assay System (Promega), as described previously^47^. Luciferase activity in untreated cells was arbitrarily given a value of 1 (after normalization to the *Renilla* luciferase signal). Luminescent signal was measured using a Centro LB960 luminometer (Berthold Technologies, Bad Wildbad, Germany).

### Silencing of *CLOCK* expression using small-hairpin RNA (shRNA)

HaCaT keratinocytes were infected with shRNA lentiviral particles (MISSION^®^ shRNA; Sigma-Aldrich) expressing scrambled (SHC203V; shCont) or *CLOCK* shRNA (TRCN0000018978; shClock), following the manufacturer’s instructions. Twenty-four hours after infection, puromycin (2 μg/mL) was added for the selection of infected cells. After 2 weeks, cells were collected, and the stable silencing of *CLOCK* expression was determined by RT-PCR.

### Statistical analysis

Statistical analysis was analyzed by one-way analysis of variance (ANOVA) followed by Sidak’s multiple comparisons test, using GraphPad Prism version 7.0 software (GraphPad Software Inc., La Jolla, CA, USA). A *P* value less than 0.05 was considered statistically significant.

### Data availability

All data generated or analysed during this study are included in this published article (and its Supplementary Information files).

## Electronic supplementary material

Supplementary Information
