# Supplementary material for: Agerarin, identified from Ageratum houstonianum, stimulates circadian CLOCK-mediated aquaporin-3 gene expression in HaCaT keratinocytes
Source: Sci Rep. 2017 Sep 11;7:11175. doi: 10.1038/s41598-017-11642-x (PMC5593932; doi:10.1038/s41598-017-11642-x)
Supplement: Supplementary file 1 — Supplementary Information [file 41598_2017_11642_MOESM1_ESM.pdf]

[Supplementary Information]

Agerarin, identified from *Ageratum houstonianum,* stimulates circadian CLOCK-mediated aquaporin-3 gene expression in HaCaT keratinocytes

Soon Young Shin[1,2], Da Hyun Lee[1], Ha-Na Gil[2], Beom Soo Kim[3], Jeong-Sook Choe[4], Jung-Bong Kim[4], Young Han Lee[1,2,*], Yoongho Lim[3,*]

[1] Department of Biological Sciences, Sanghuh College of Life Sciences, Konkuk University, Seoul 05029, Republic of Korea

[2] Cancer and Metabolism Institute, Konkuk University, Seoul 05029, Republic of Korea

[3] Division of Bioscience and Biotechnology, BMIC, Konkuk University, Seoul 05029, Republic of Korea

[4] Department of Agrofood Resources, National Academy of Agricultural Sciences, Rural Development Administration, Jeonju 55365, Republic of Korea

[*]Correspondence and requests for materials should be addressed to Y.H.L. (E-mail: yhlee58@konkuk.ac.kr) or Y.L. (E-mail: yoongho@konkuk.ac.kr)

Supplemental Table S1

Table S1. The complete assignments of the ^{1}H and ^{13}C NMR data of AG-H1.

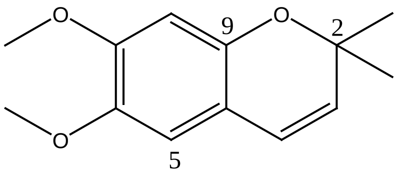

number	δ of ^{13}C /ppm	Multiplicity*	δ of 1H / ppm
7	149.9	s	-
9	147.4	s	-
6	143.3	s	-
3	128.4	d	5.44(d, 9.7)
4	122.1	d	6.20(d, 9.7)
10	113.2	s	-
5	110.0	d	6.50(s)
8	101.2	d	6.38(s)
2	76.2	s	-
6-OMe	56.7	q	3.78(s)
7-OMe	56.1	q	3.80(s)
2-CH_3	27.8	q	1.38(s)

* Multiplicities s, d, and q denote singlet, doublet, and quartet, respectively.

Supplemental Figure S1

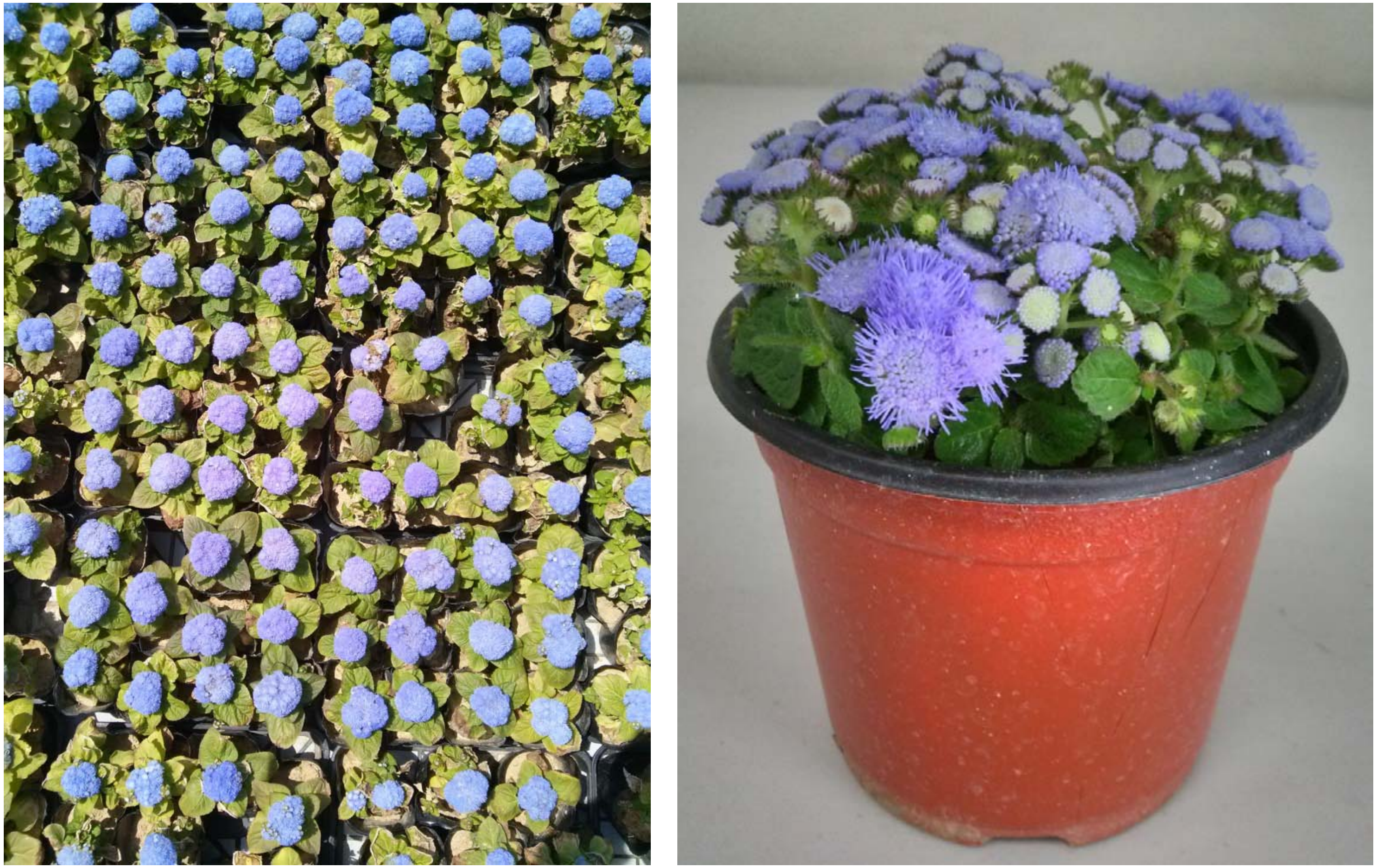

Figure S1. *A. houstonianum* grown in flowerpots.

Supplemental Figure S2

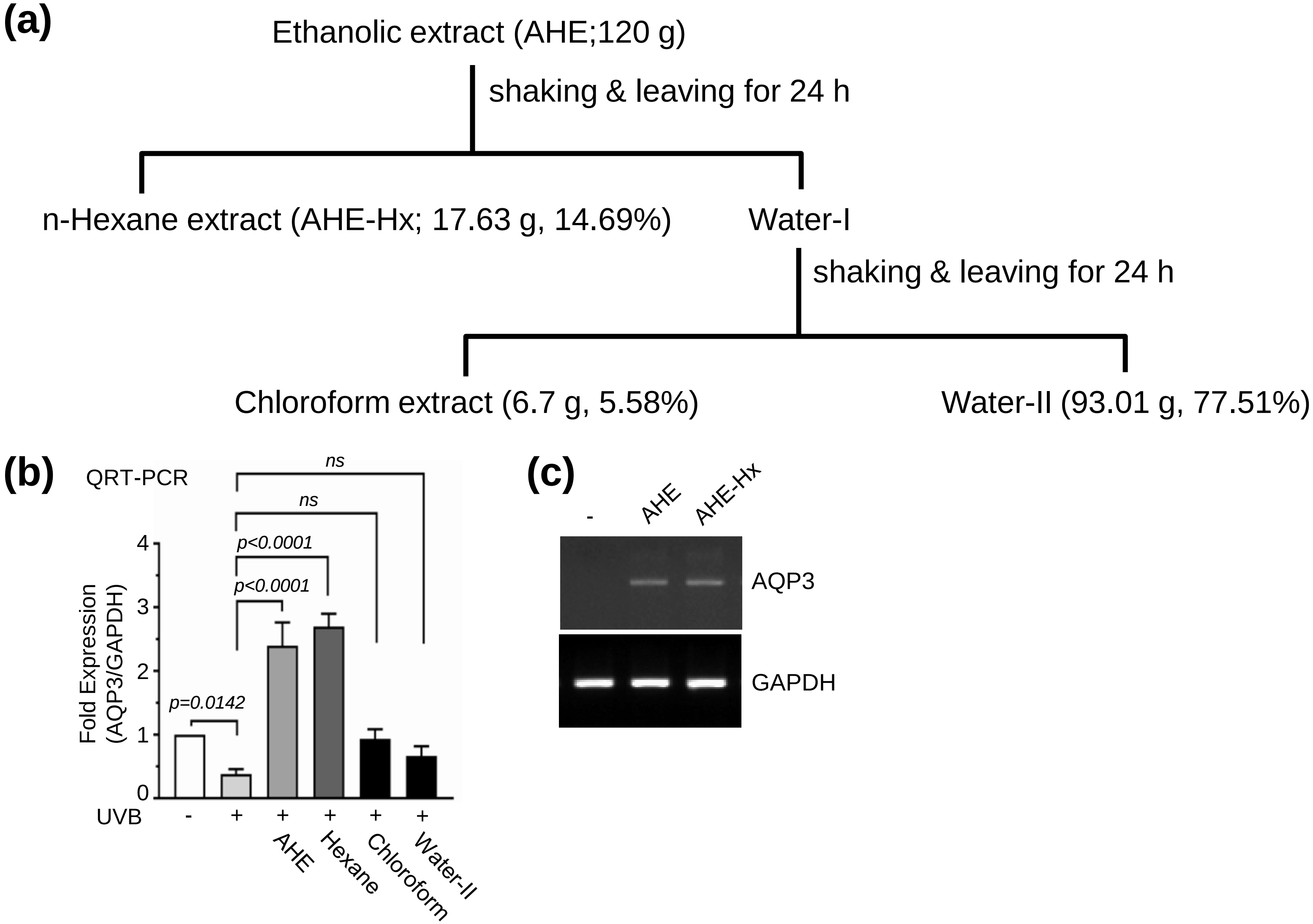

Figure S2. Isolation of active components from A. *houstonianum* ethanolic extract. (a) Fractionation scheme for the isolation of active components. (b) recovering of UVB-induced suppression of AQP3 expression by *A*. *houstonianum* fractions, and (c) inducing of AQP3 mRNA expression .

Supplemental Figure S3

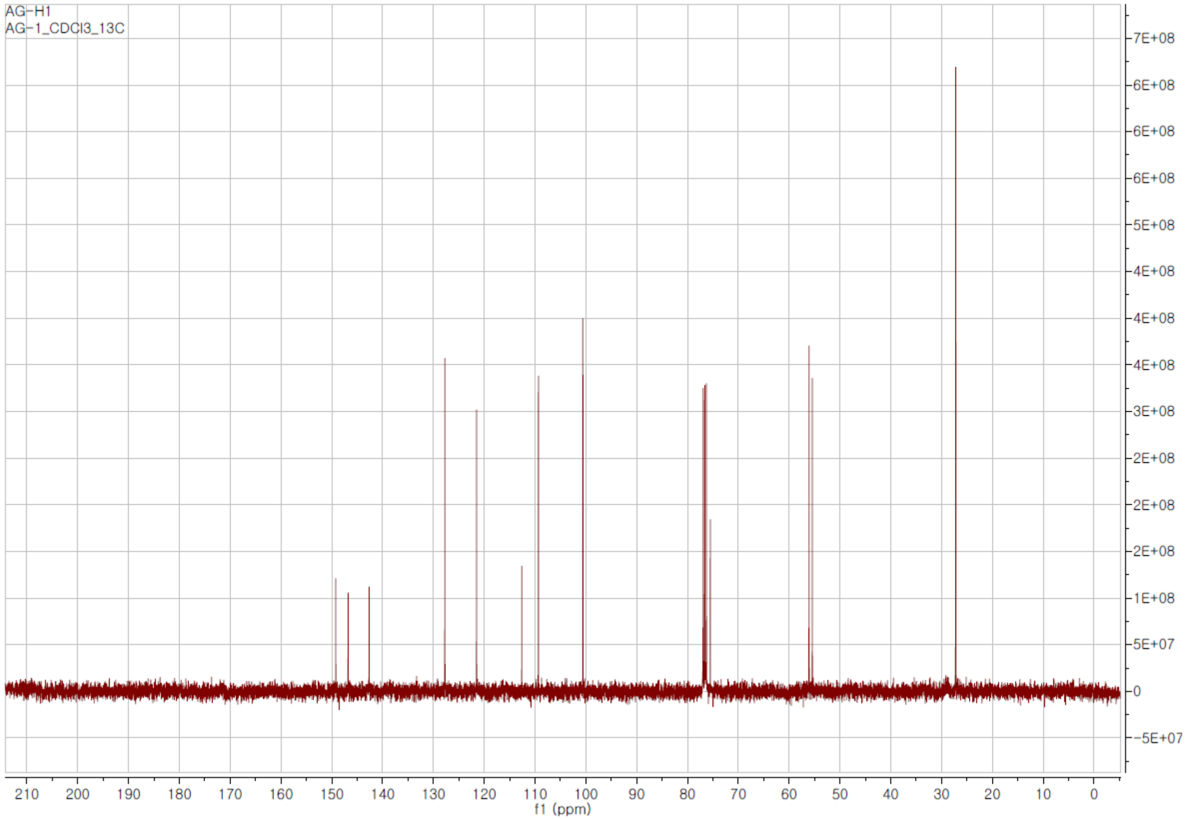

Figure S3. The ^{13}C NMR spectrum of AG-H1.

Supplemental Figure S4

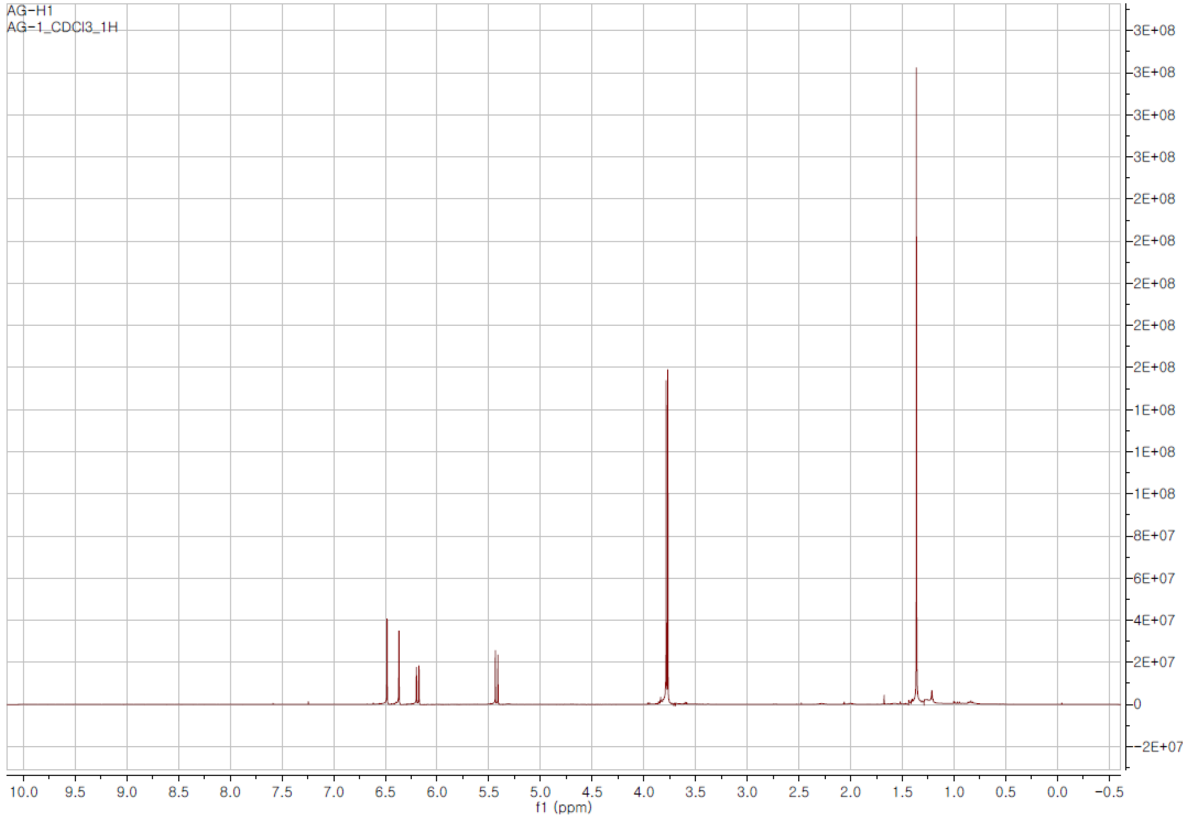

Figure S4. The ^{1}H NMR spectrum of AG-H1.

Supplemental Figure S5

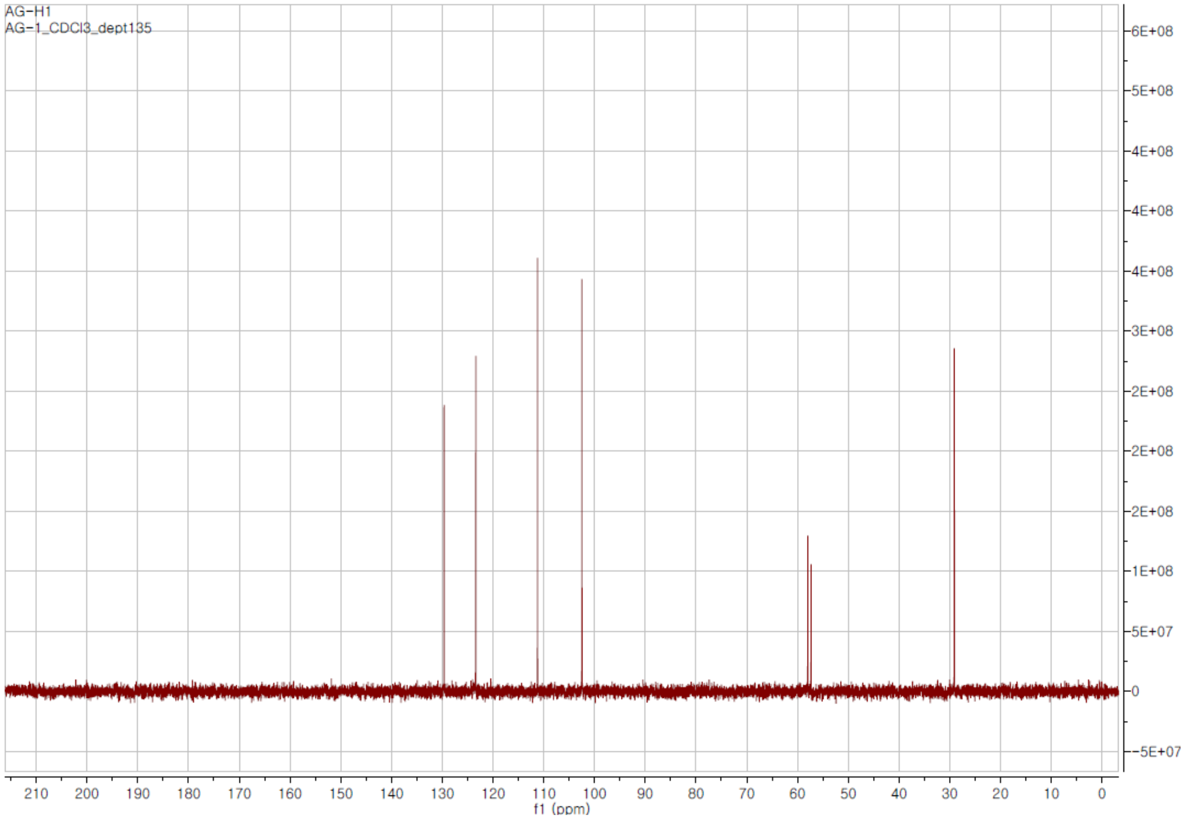

Figure S5. The DEPT spectrum of AG-H1.

Supplemental Figure S6

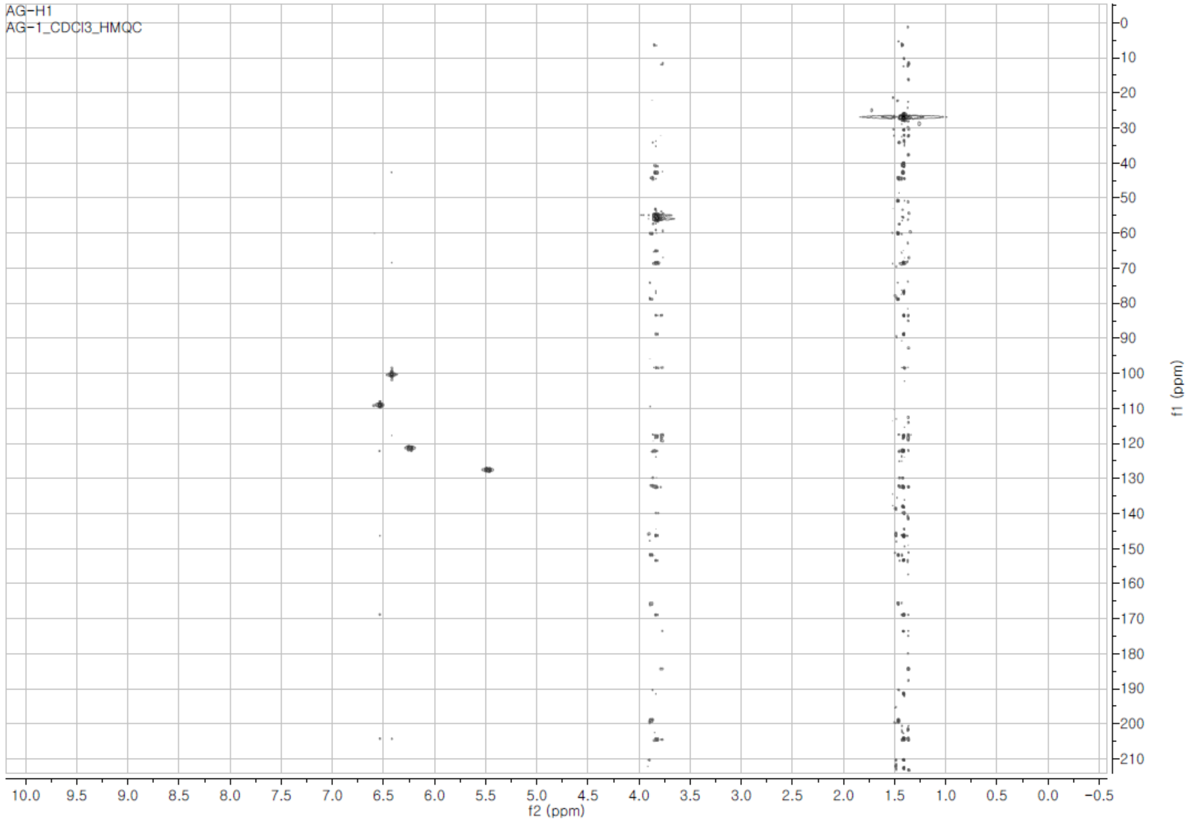

Figure S6. The HMQC spectrum of AG-H1.

Supplemental Figure S7

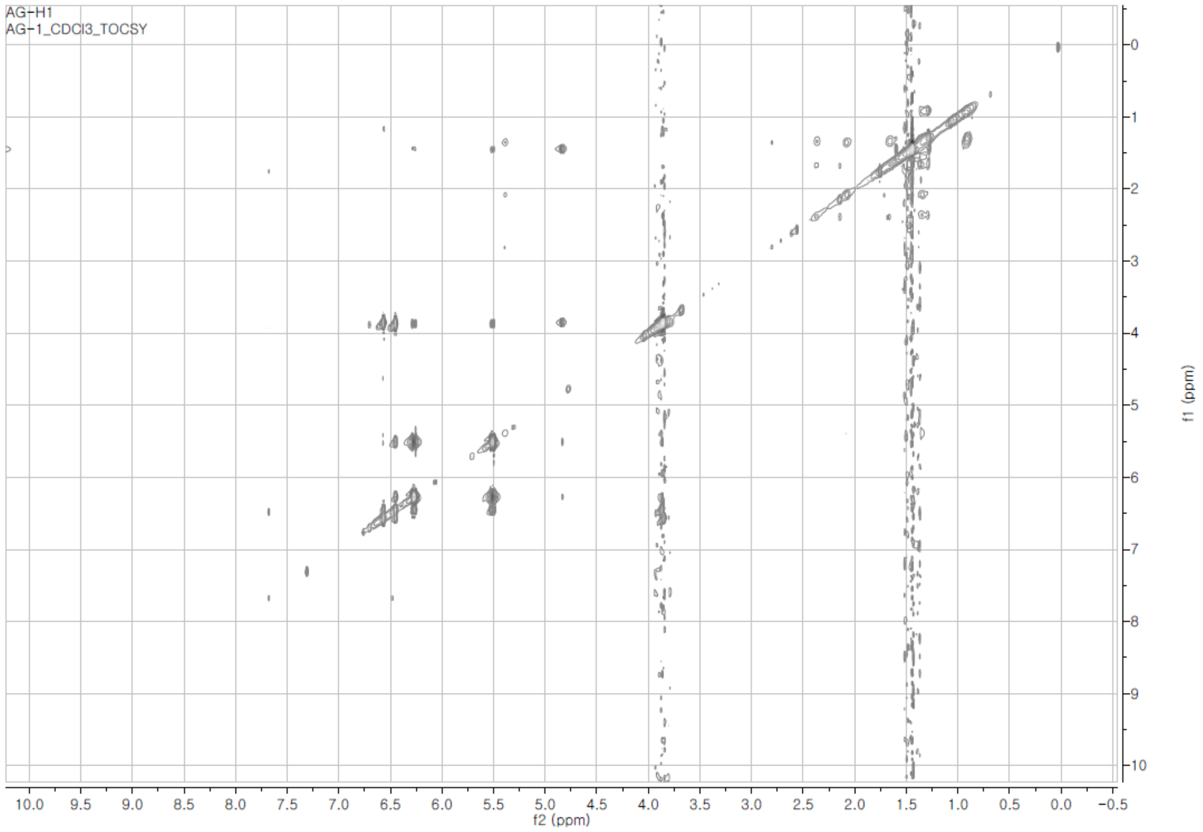

Figure S7. The TOCSY spectrum of AG-H1.

Supplemental Figure S8

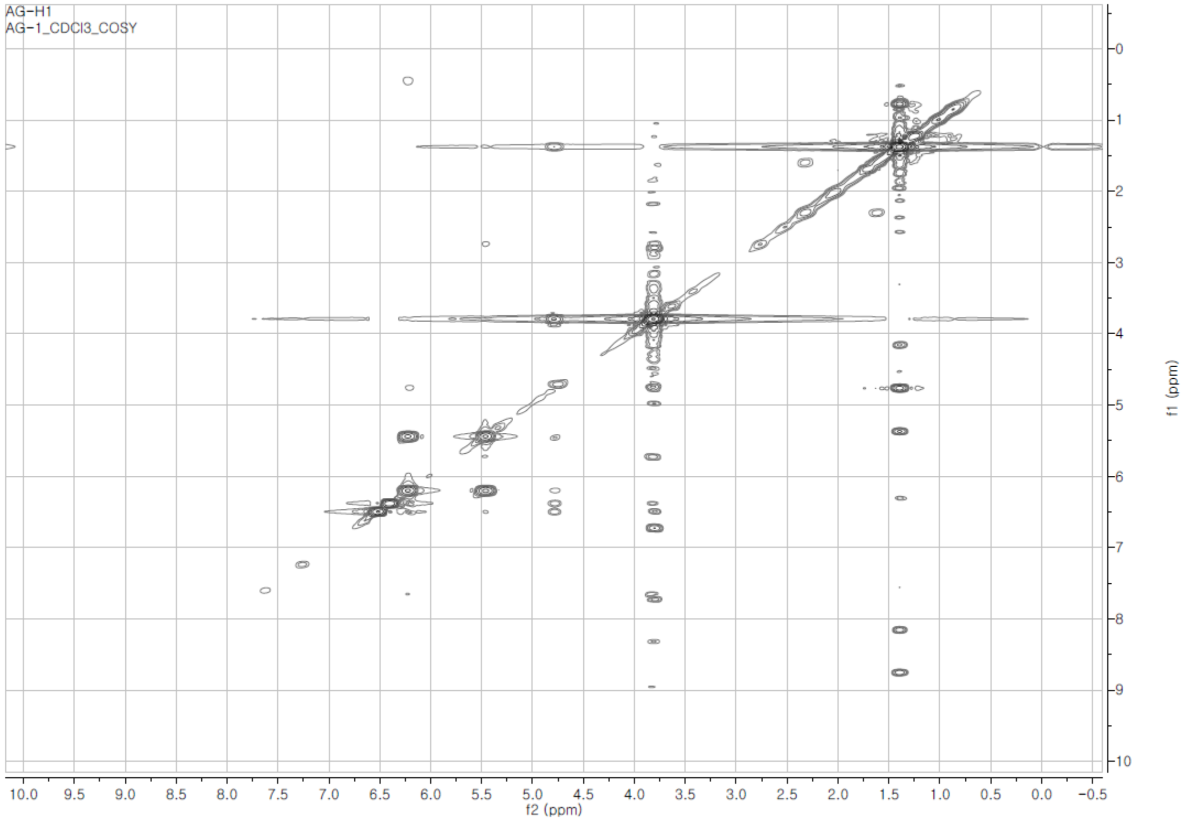

Figure S8. The COSY spectrum of AG-H1.

Supplemental Figure S9

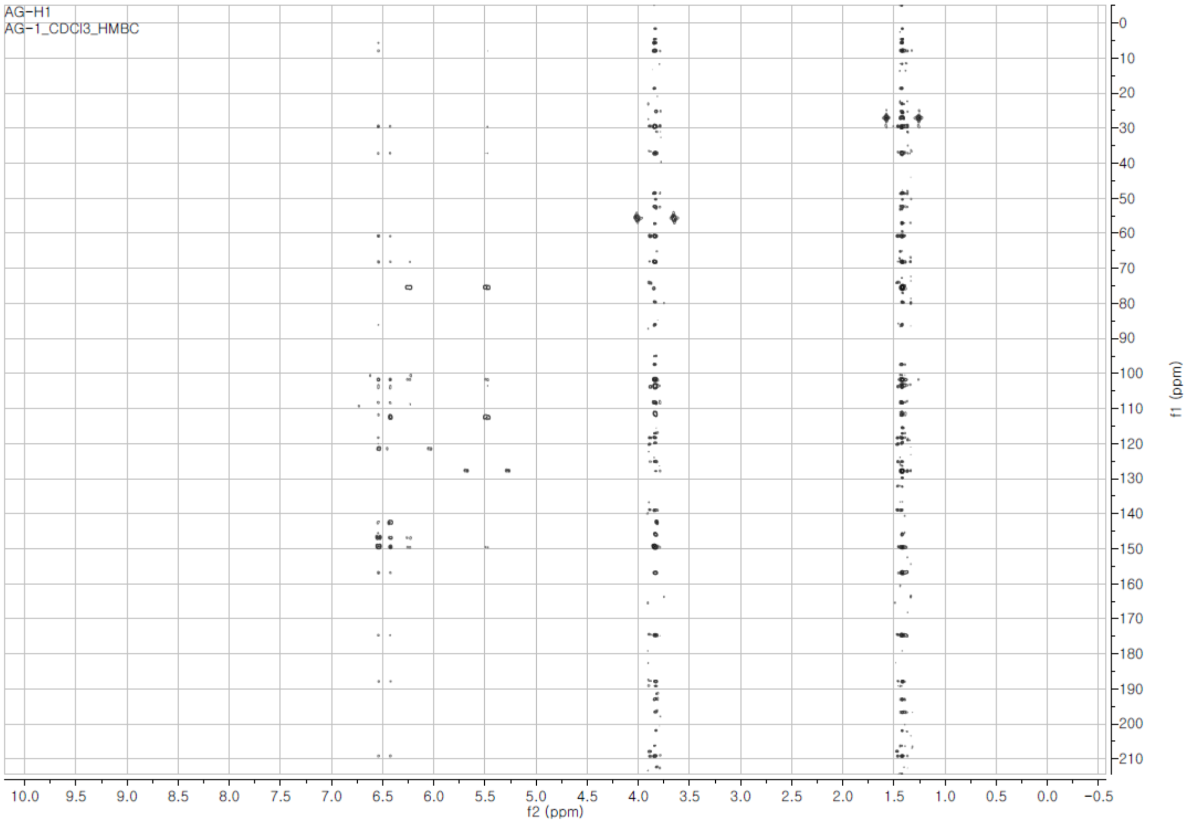

Figure S9. The HMBC spectrum of AG-H1.

Supplemental Figure S10

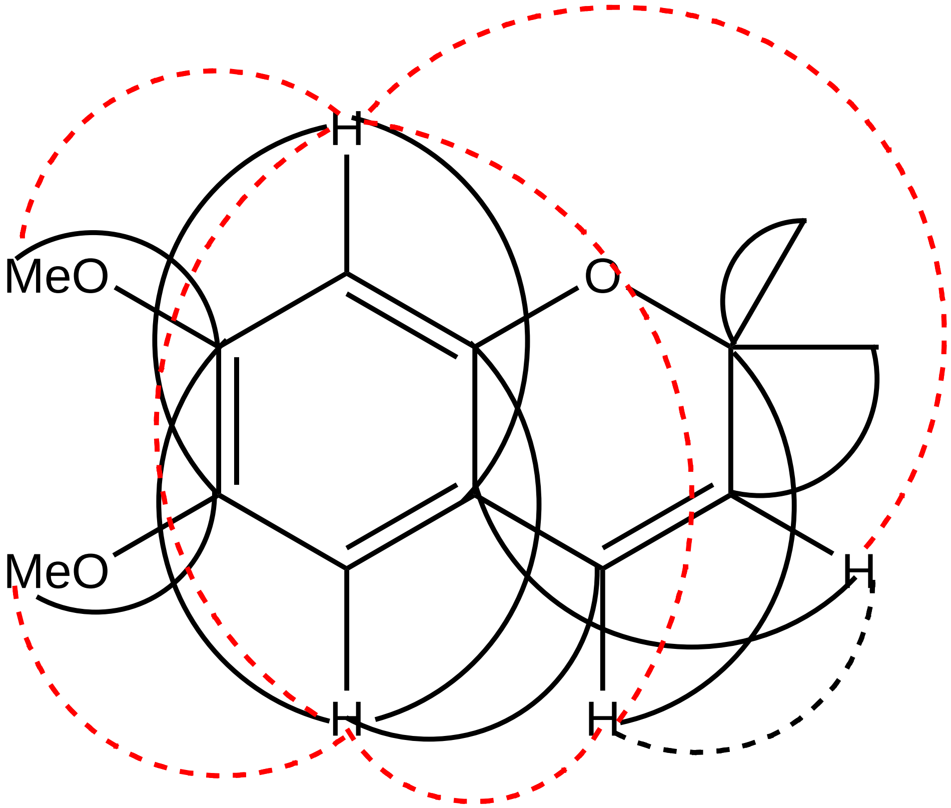

Figure S10. The important correlations obtained from the interpretation of COSY (dot lines) and HMBC (solid lines).

Supplemental Figure S11

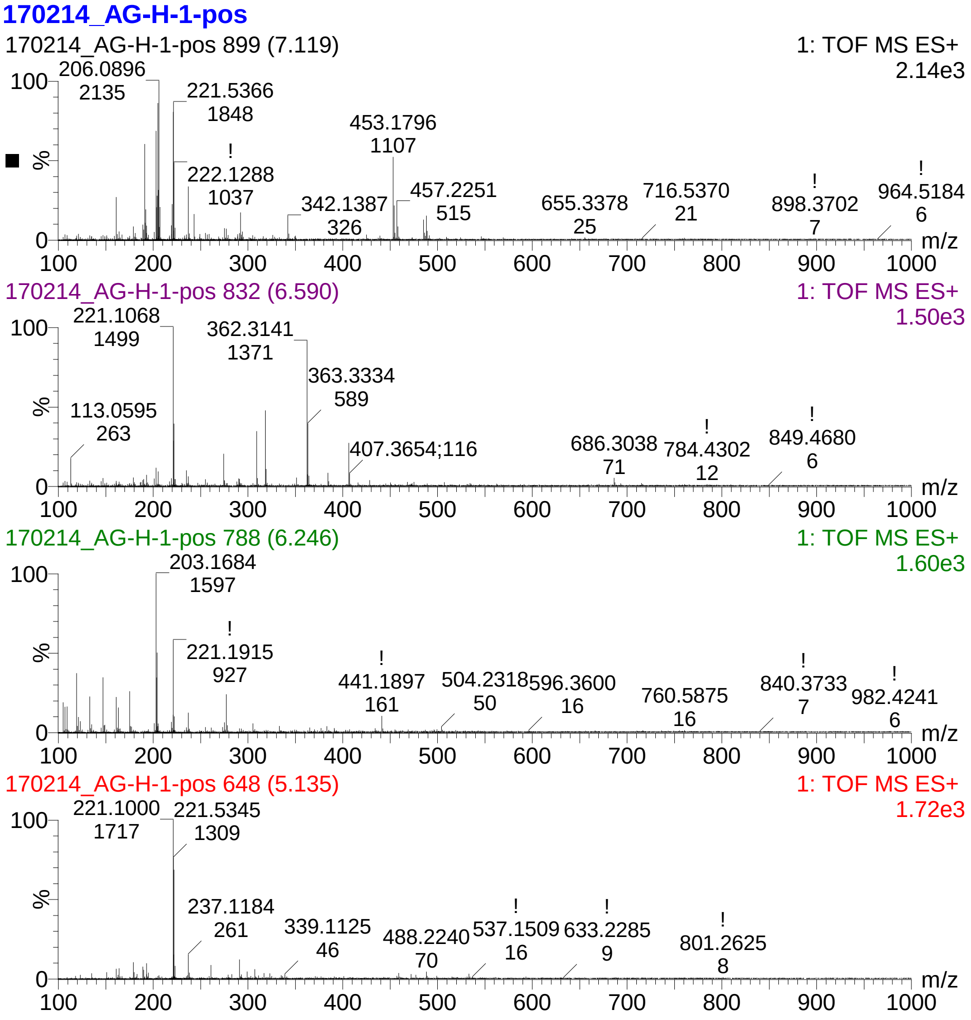

Figure S11. The high-resolution mass spectrum of AG-H1 obtained from ultra-performance liquid chromatography-hybrid quadrupole-time-of-flight mass spectrometry.

Supplemental Figure S12

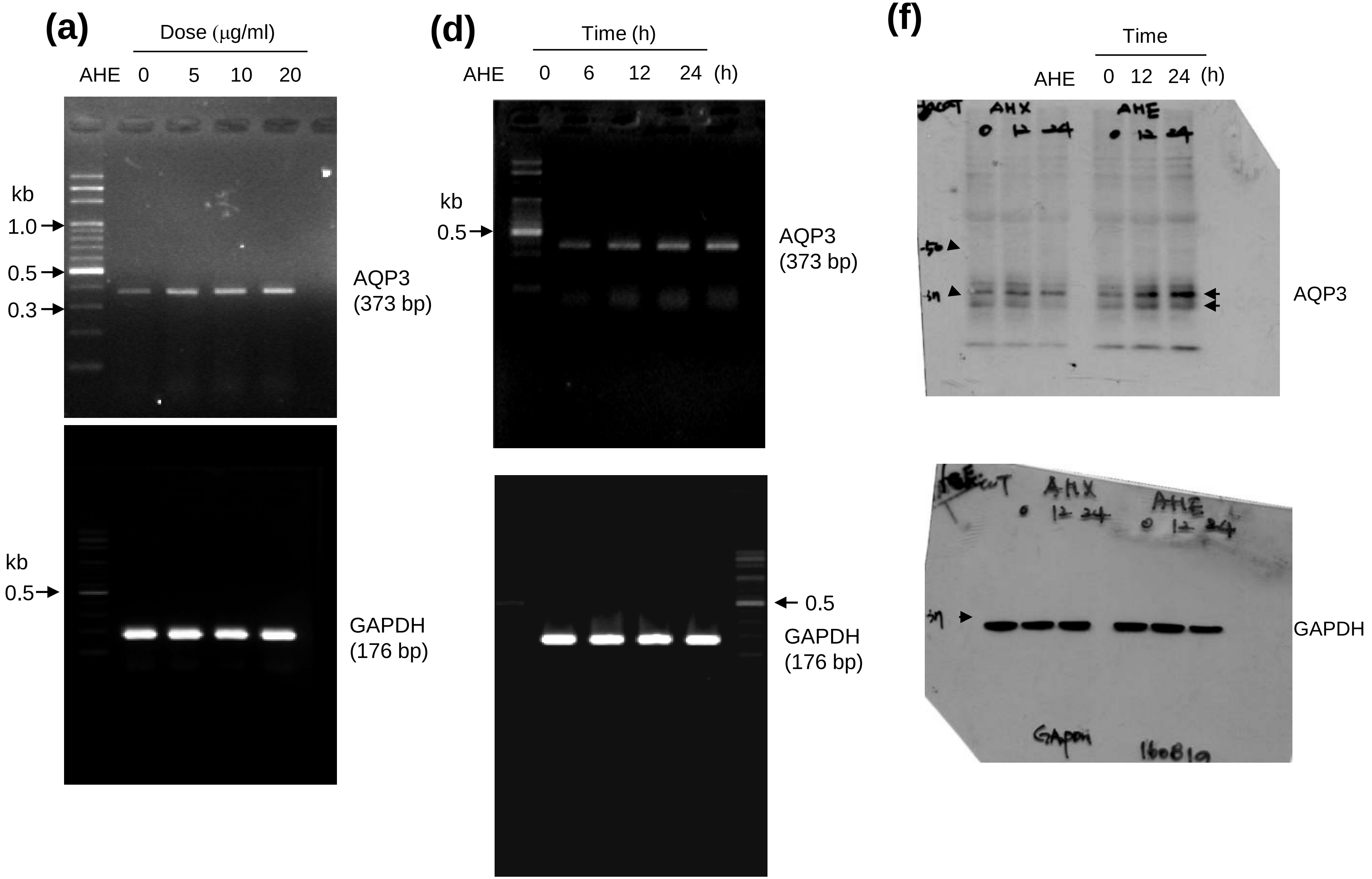

Figure S12. Full-length gels and blots in Figure 1(a), (d), and (f).

Supplemental Figure S13

(a)

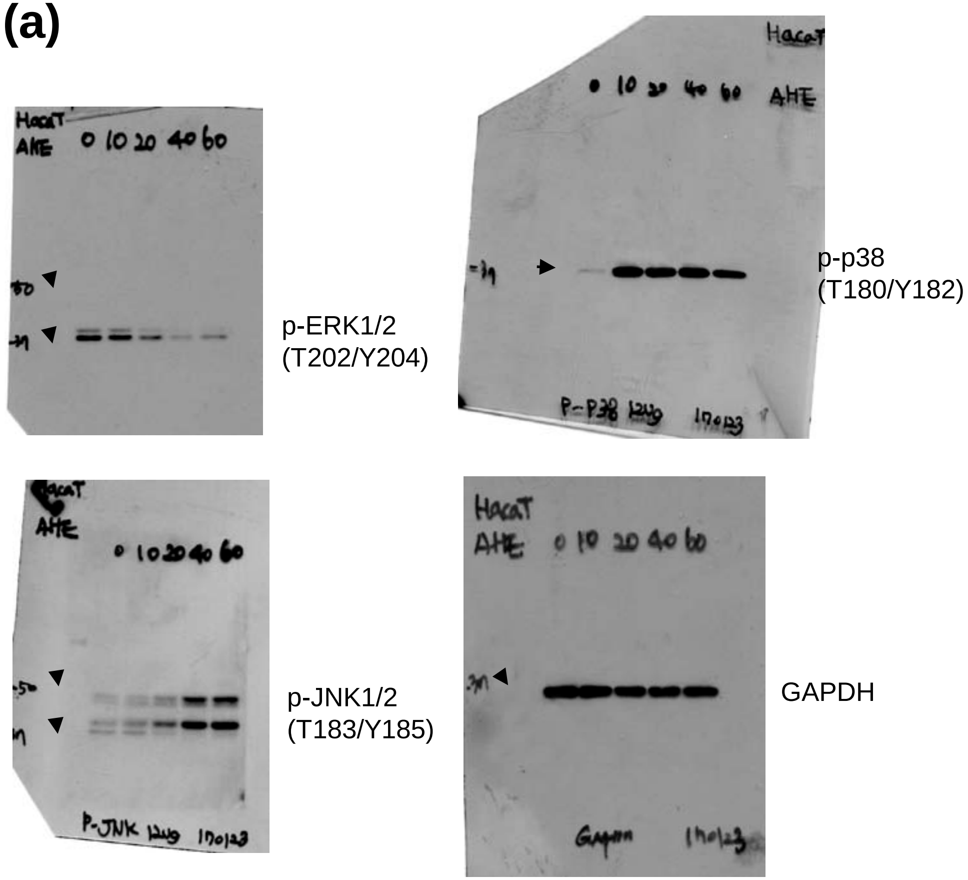

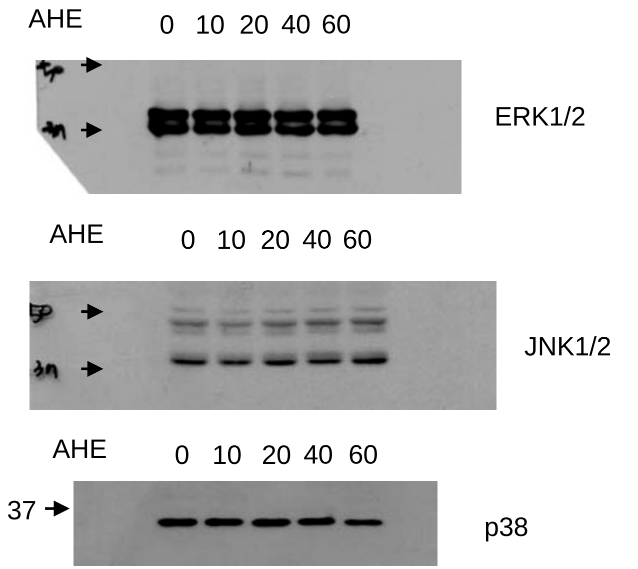

(b)

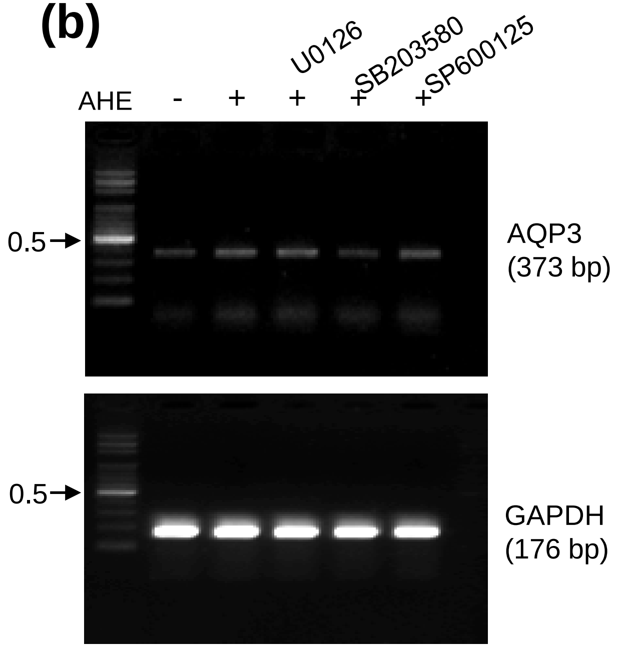

Figure S13. Full-length gels and blots in Figure 2(a) and (b).

Supplemental Figure S14

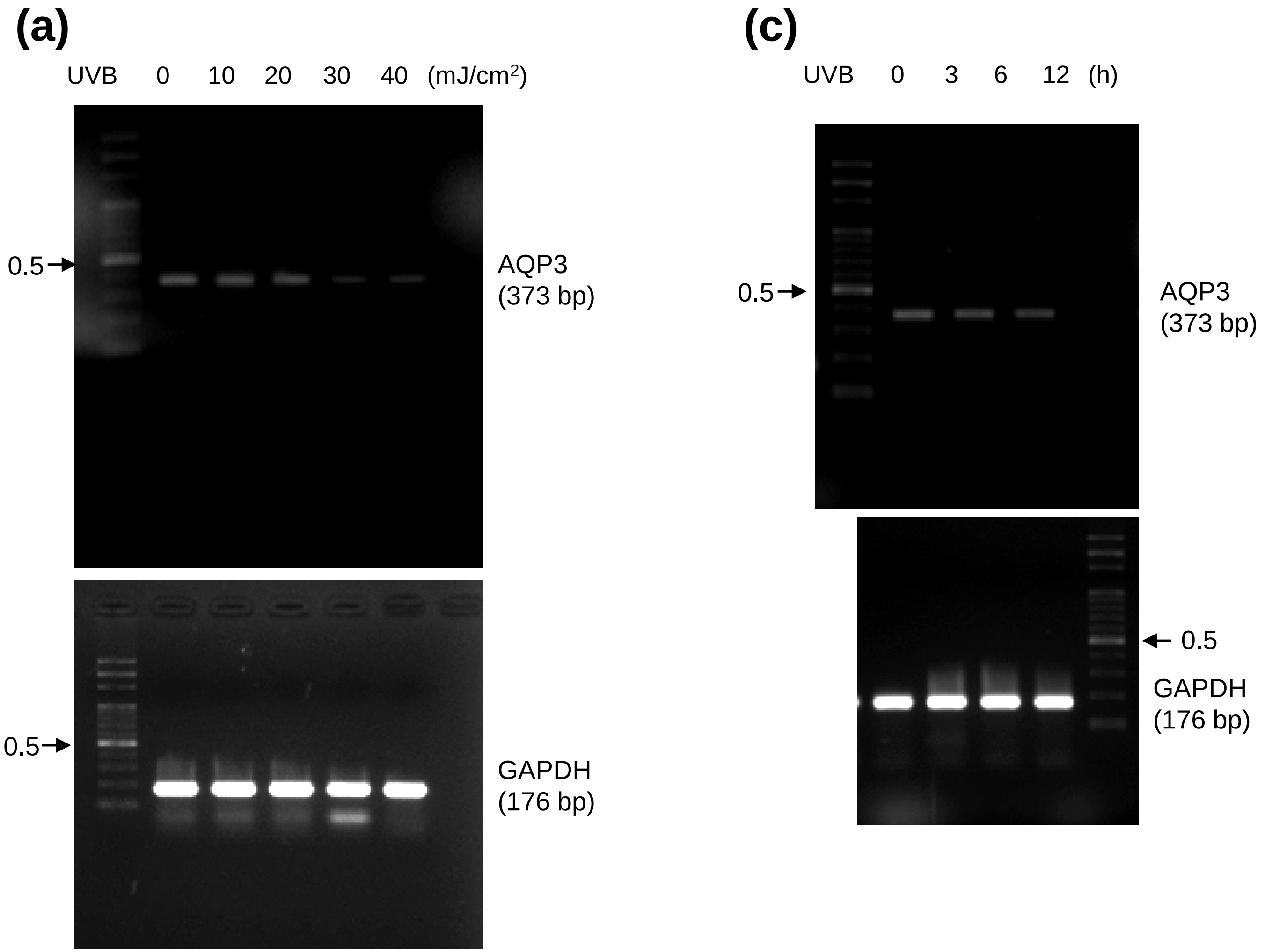

Figure S14. Full-length gels in Figure 3(a) and (c).

Supplemental Figure S15

(a)

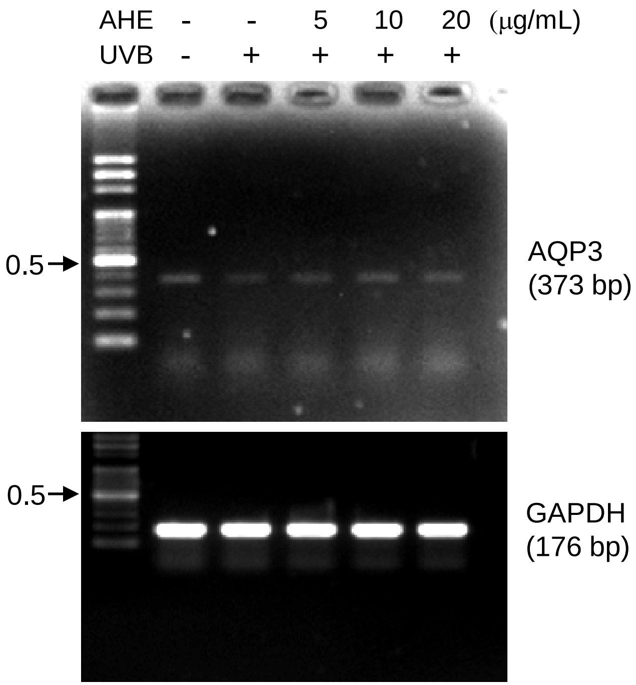

(d)

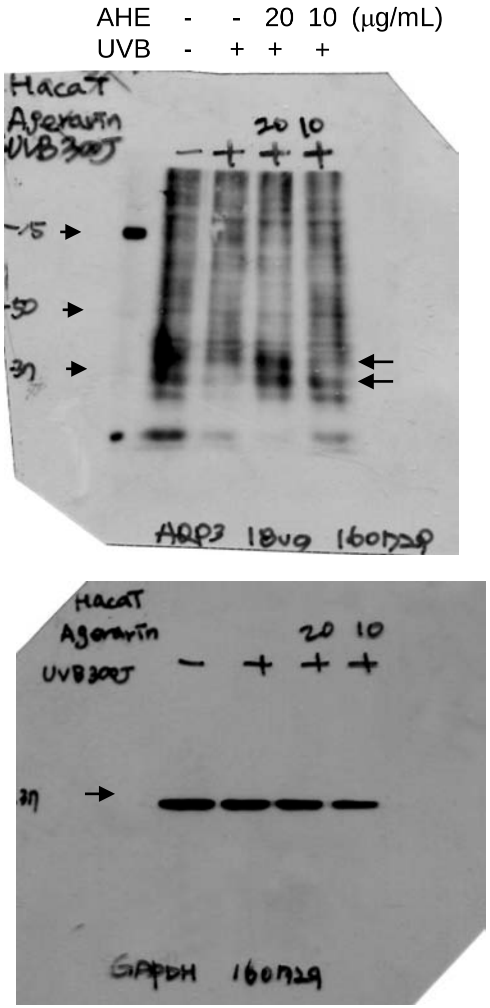

Figure S15. Full-length gels and blots in Figure 4(a) and (d).

Supplemental Figure S16

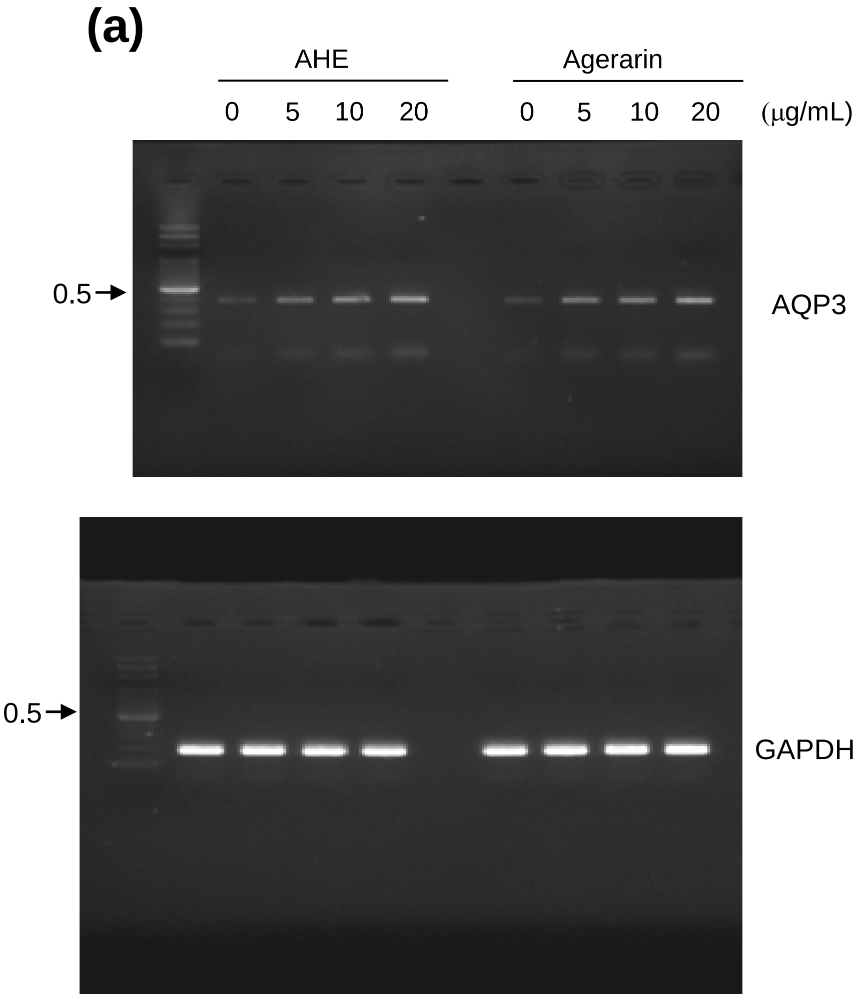

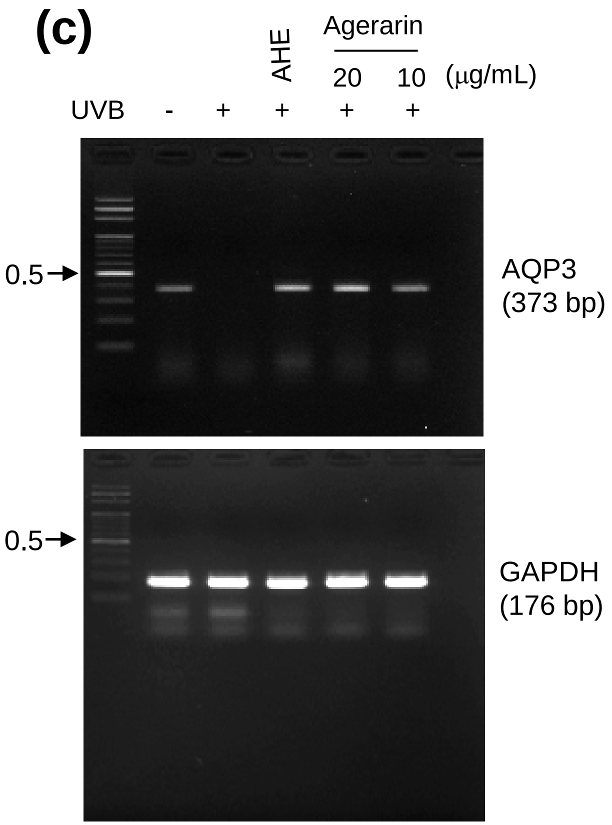

Figure S16. Full-length gels in Figure 6(a) and (c).

Supplemental Figure S17

(b)

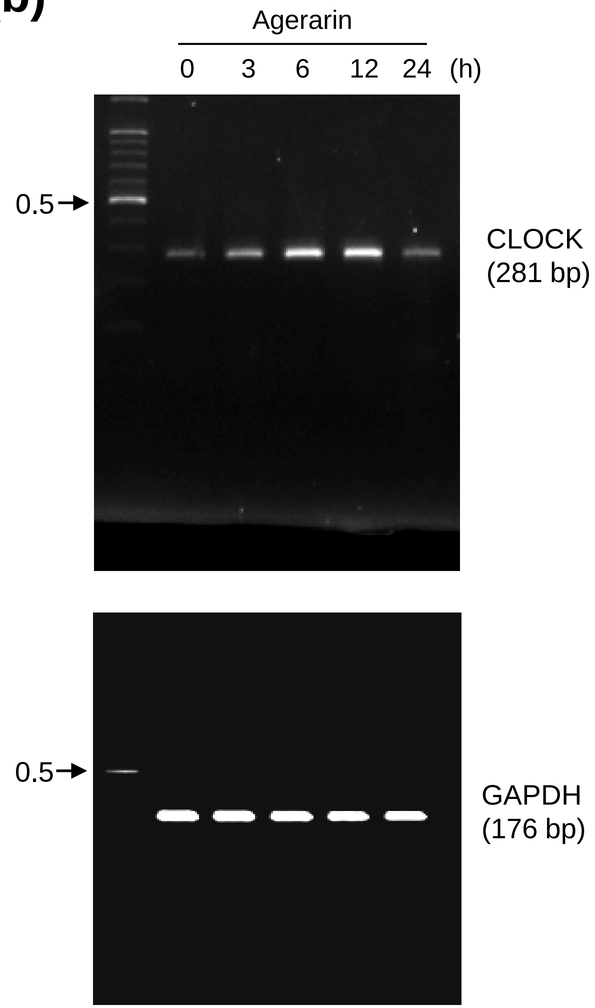

(d)

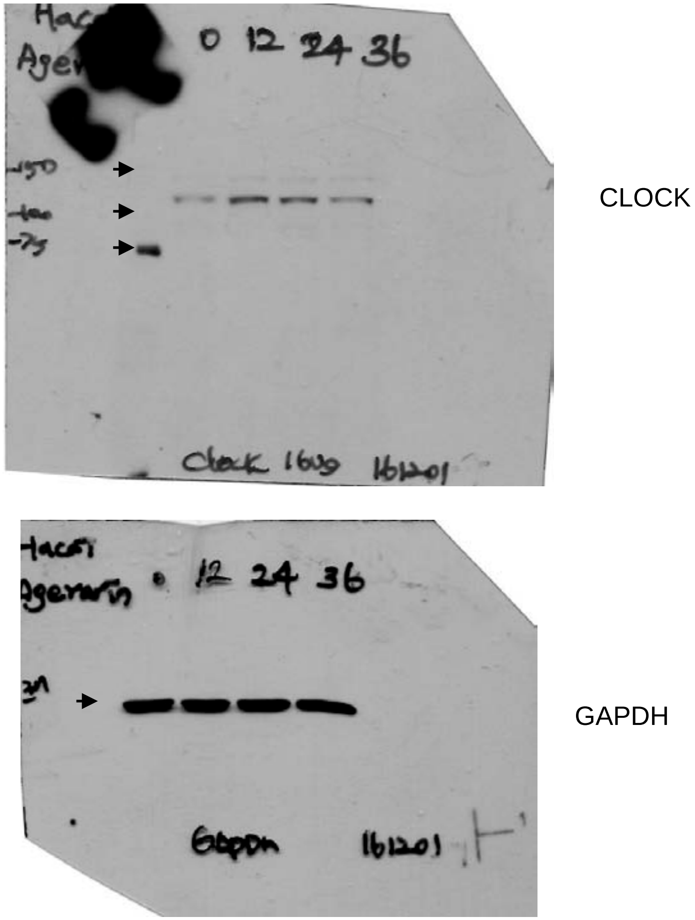

Figure S17. Full-length gels and blots in Figure 7(b) and (d).

Supplemental Figure S18

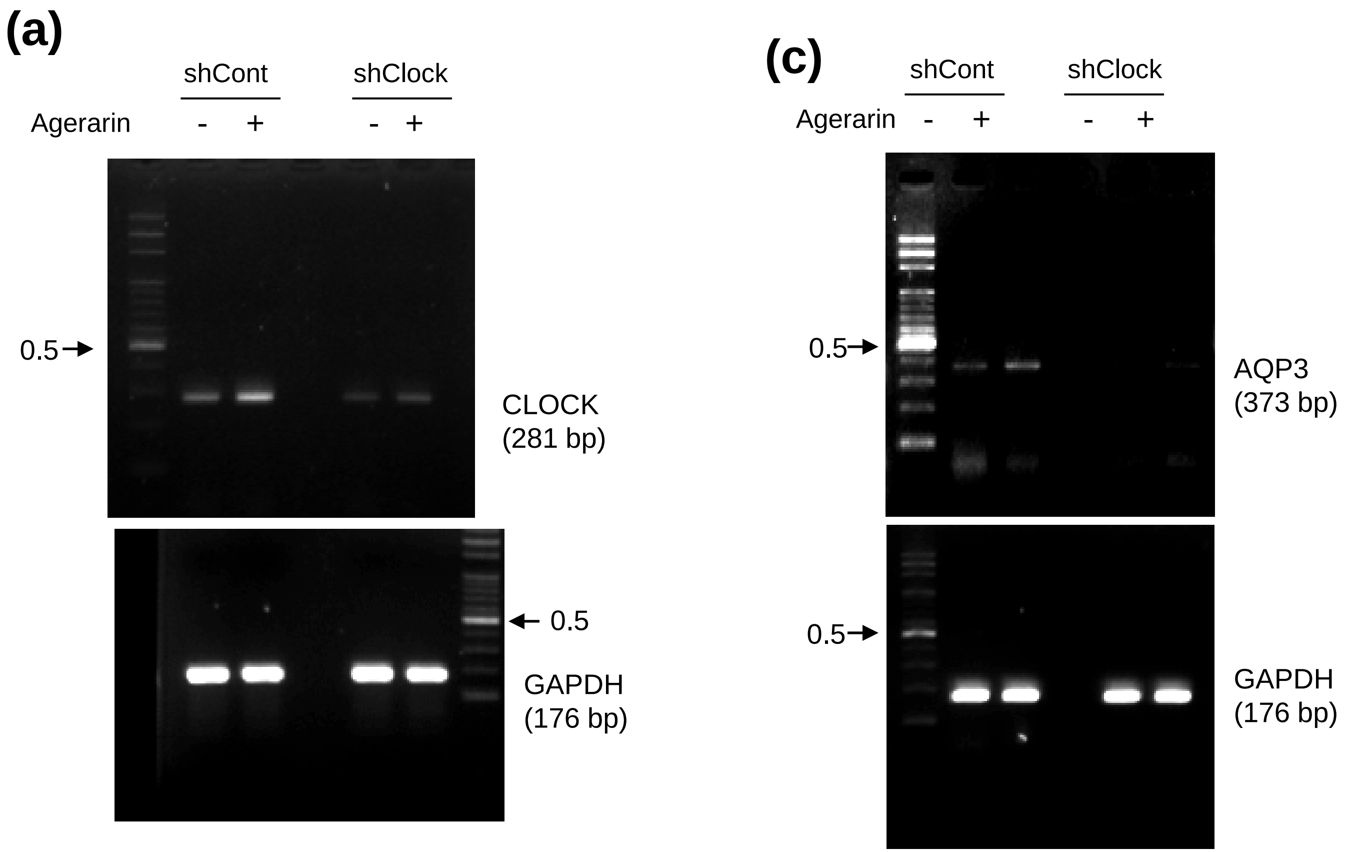

Figure S18. Full-length gels in Figure 8(a) and (c).
